# Systematic Material Optimization for Membrane Distillation Resource Recovery through Materials Informatics, Life Cycle Assessment, and Industrial Scalability

**DOI:** 10.1021/acsestengg.6c00062

**Published:** 2026-04-10

**Authors:** Saketh Merugu, Keval Bharatbhai Suthar, Anju Gupta

**Affiliations:** Department of Mechanical, Industrial and Manufacturing Engineering, 7923 The University of Toledo, 2801 West Bancroft Street, Toledo, Ohio 43606, United States

**Keywords:** Ansys, resource recovery, circular water economy, desalination, sustainability

## Abstract

Material selection for membrane distillation (MD) remains dominated by empirical trial-and-error. This study presents the first application of Ansys Granta materials informatics to thermally driven membrane separation, integrating database-driven screening, direct contact MD (DCMD) experimental validation, and life cycle assessment (LCA) to identify optimal membrane materials across diverse circular water economy contexts. Twenty-two candidates spanning polymers, biopolymers, and ceramics were evaluated against thermal and mechanical performance, vapor transport efficiency, and chemical compatibility across five aggressive feed environments. LCA at a representative 10,000 m^3^·day^–1^ facility scale reveals a counterintuitive lifecycle inversion: PEEK and PES, the two highest production-phase energy materials among all 22 candidates achieving net-positive lifecycle sustainability through robust end-of-life recycling, demonstrate that the production carbon footprint is a misleading proxy for environmental performance, a finding with implications beyond membrane engineering. Three commercial membranes—polypropylene (PP), polyvinylidene fluoride (PVDF), and polytetrafluoroethylene (PTFE)—validated the informatics predictions through DCMD desalination testing, achieving fluxes of 14 ± 2, 11 ± 3, and 29 ± 4 kg·m^–2^·h^–1^ with >99% salt rejection. Predicted flux agreed closely (R^2^ ≈ 1); styrene–butadiene–styrene (SBS) exhibited the highest theoretical flux (413 kg·m^–2^·h^–1^), a theoretical upper bound reflecting intrinsic vapor transmission rather than practical MD performance. Cross-property analysis identified maximum service temperature and tensile strength as the strongest correlated pair (*r* = 0.67). The multicriteria performance index (Π) reveals fundamentally context-dependent rankings: titania leads under balanced weighting (Π = 0.67), SBS under flux priority (Π = 0.77), and PVC under sustainability priority (Π = 0.77). No universally optimal material exists; this replicable framework replaces single-criterion optimization with transparent, application-specific material guidance for circular water economy MD deployment.

## Graphical abstract

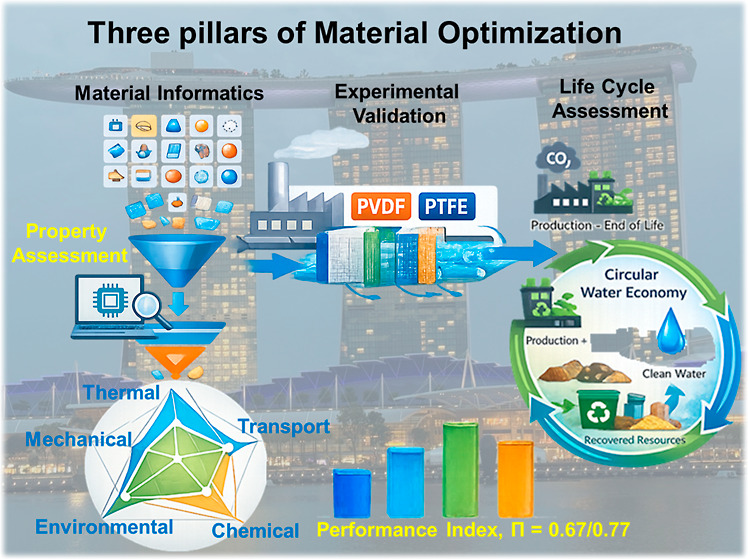

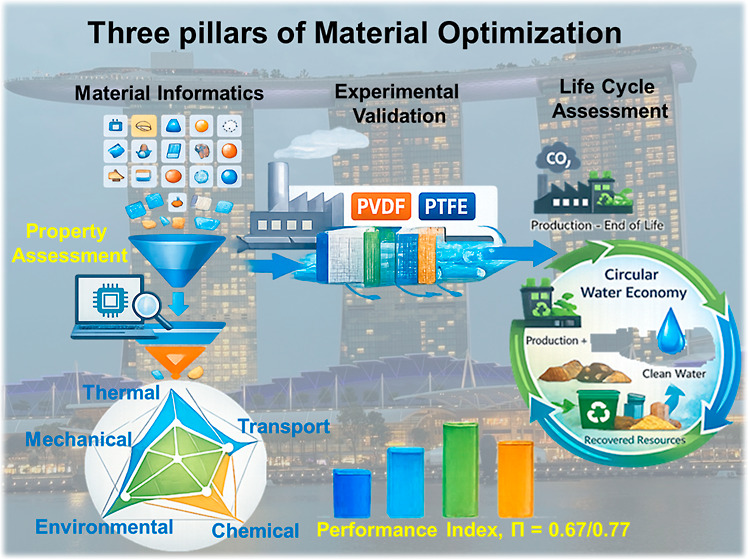

## 1 Introduction

The global freshwater crisis, driven by population growth, industrial expansion, and climate-induced water stress, necessitates a significant advancement from linear “extract–use–discard” water management to circular water economy approaches that simultaneously recover valuable resources and produce clean water for reuse.^1,2^ Current linear water systems lead to freshwater depletion and loss of recoverable minerals, nutrients, and organic matter embedded within wastewater streams.^3–5^ The transition to a circular water economy requires integrated systems that recover resources from wastewater while producing water suitable for reuse or safe discharge.^6–8^

Membrane distillation (MD) has emerged as a transformative separation platform enabling this circular economic vision.^8^ As a thermally driven membrane process, MD employs hydrophobic membranes to facilitate selective vapor transport across temperature gradients, separating volatile water molecules from nonvolatile dissolved solutes, salts, and organic species.^9^ Unlike pressure-driven membrane processes that separate based on size or charge, MD operates on volatility differences, offering distinctive advantages for selective resource recovery from chemically diverse and thermally challenging waste streams.^10^ These attributes directly position MD as an environmentally beneficial technology: by operating at lower pressures than reverse osmosis and utilizing low-grade or waste thermal energy, MD reduces operational energy demand while enabling treatment of high-salinity and chemically aggressive waste streams that conventional technologies cannot efficiently process, thereby reducing pollutant discharge to receiving water bodies. MD’s reduced fouling susceptibility, minimal chemical pretreatment requirements, and operational flexibility across diverse temperature ranges position it as an enabling technology for resource recovery applications.^11,12^ The central premise of this work is that optimal membrane material selection is inherently context-dependent and requires a multicriteria framework that simultaneously considers technical performance, chemical compatibility, and environmental sustainability, dimensions that single-criterion optimization approaches cannot adequately address. Within the circular water economy context, MD demonstrates exceptional utility across diverse sectors, such as semiconductor and advanced manufacturing industries,^13,14^ food and beverage industries,^15^ and mining and hydrometallurgical sectors.^16,17^ Despite MD’s substantial promise for circular water economy applications, systematic material selection frameworks that simultaneously optimize technical performance, chemical compatibility across diverse industrial wastewaters, environmental sustainability through full life cycle assessment (LCA), and economic viability remain absent from the literature.^18,19^ From an environmental science perspective, MD’s capacity to treat diverse and aggressive waste streams positions it as a key technology for reducing environmental pollutant discharge, recovering critical materials that would otherwise require energy-intensive primary extraction, and closing the water-resource loop in industrial ecosystems. The environmental benefit extends beyond water purification to encompass reduced freshwater extraction pressure, recovery of minerals critical for clean energy technologies such as lithium, cobalt, and rare earth elements, and minimization of concentrated brine disposal, a significant contributor to aquatic ecosystem degradation. Existing approaches to membrane material discovery rely predominantly on empirical trial-and-error methodologies or single-criterion optimization that fails to capture the complex multidimensional trade-offs inherent in industrial deployment decisions. Recent advances demonstrate that integrating informatics-driven screening with experimental validation can accelerate functional membrane design, with machine learning approaches correlating structural features with separation performance in complex systems, providing frameworks transferable to water purification and MD applications.^20^

This study integrates Ansys Granta materials informatics, DCMD experimental validation, and LCA to systematically screen 22 candidate membrane materials, spanning polymers, biopolymers, and ceramics across thermal stability, mechanical robustness, chemical compatibility in five aggressive feed environments, vapor transport, and full lifecycle environmental burden for a representative 10,000 m^3^·day^–1^ facility. Four contributions follow: (i) the first replicable multicriteria material selection framework for MD, combining informatics with experimental validation and LCA; (ii) a weighting-scenario framework quantifying how rankings shift across flux-priority, sustainability-priority, and balanced operational contexts; (iii) evidence that end-of-life recovery pathways can reverse production-phase sustainability rankings; and (iv) comprehensive chemical compatibility mapping enabling application-specific material guidance. Three research questions structure the work: whether database-derived bulk properties reliably predict relative MD performance; how multidimensional trade-offs reshape material rankings under different operational priorities; and whether high production-phase burdens necessarily indicate unsustainable material choices.

The chemical versatility of MD materials becomes critical when considering the extreme pH environments encountered across diverse industrial sectors. For instance, pulp and paper manufacturing generates caustic alkaline effluents (pH > 13) during pulping operations, while mineral processing and metal finishing industries produce strongly acidic waste streams (pH < 3) from acid leaching and pickling operations. These chemically aggressive environments demand membrane materials demonstrating exceptional resistance across the full pH spectrum requirement that conventional pressure-driven membrane processes often cannot satisfy due to greater sensitivity to pH-induced membrane degradation and fouling. MD’s operation based on volatility-driven separation rather than size exclusion offers inherent advantages for extreme pH applications, provided the underlying membrane material maintains chemical stability across these conditions. Consequently, systematic assessment of material compatibility across both alkaline and acidic environments represents a critical dimension of material selection for comprehensive circular economic water implementation spanning diverse industrial contexts.

The key contributions of this study are (i) the first documented application of Ansys Granta materials informatics to thermally driven membrane separation, establishing a replicable database-driven screening methodology that integrates experimental DCMD validation and LCA across 22 candidate materials spanning polymers, biopolymers, and ceramics, a scope and platform combination with no prior precedent in the MD or broader membrane separation literature; (ii) a weighting-scenario framework that quantifies how material rankings shift across flux-priority, sustainability-priority, and balanced operational contexts to provide application-specific guidance in place of single-criterion recommendations; (iii) evidence that lifecycle environmental assessment incorporating end-of-life recovery can reverse production-phase sustainability rankings; and (iv) comprehensive chemical compatibility mapping across five aggressive feed environments enabling application-specific material guidance. Specifically, this study addresses three interconnected research questions: (1) Can materials informatics database-derived bulk properties reliably predict relative MD performance, thereby replacing conventional trial-and-error material screening? (2) How do multidimensional material properties such as thermal stability, mechanical strength, vapor transport, chemical compatibility, and environmental sustainability interact to determine optimal material selection under different operational priorities? (3) To what extent do end-of-life recovery pathways modify lifecycle environmental assessments, and do materials with high production-phase burdens necessarily represent unsustainable choices?

## 2 Materials and Methodology

This study employed an Ansys Granta EduPack 2025 R1 (GRANTA Design, Cambridge, UK), specifically the Materials module integrated with Eco Audit functionality. The Materials module provided comprehensive property data for approximately 4700 commercial materials spanning polymers, biopolymers, and ceramics, sourced from peer-reviewed literature, manufacturer technical datasheets, and standardized testing protocols (ASTM and ISO). Data quality classifications were assigned to each property entry: A = peer-reviewed literature, B = manufacturer data, and C = estimated values, enabling transparent traceability and confidence assessment. The Eco Audit module, grounded in industry-standard life cycle inventory (LCI) databases such as ecoinvent 3.8, USDA BioPreferred, and NREL U.S. Life Cycle Inventory, enabled quantification of production-phase environmental impacts including embodied energy and greenhouse gas emissions. A hierarchical multistage filtering protocol was employed to systematically identify candidate membrane materials from the comprehensive Ansys Granta database. Figure 1 presents the complete material screening workflow, showing systematic elimination at each filtering stage. The process begins with a comprehensive database of ∼4000 materials and applies sequential filtering based on membrane-specific criteria. Stage 0: Initial classification retained 216 candidates: 168 polymers, 22 biopolymers, and 26 ceramics. The thermal stability filter (maximum service temperature ≥50 °C) eliminated 6 low-temperature elastomers. The mechanical integrity filter (tensile strength ≥5 MPa) removed 21 soft elastomers. The economic viability and commercial availability filter (production volume >1000 MT/year, cost < $50/kg) excluded 17 specialty materials. The application relevance and data completeness filter (≥80% property coverage and documented water treatment/separation history) eliminated 150 materials lacking sufficient data or prior application. The final selection comprises 22 materials spanning 17 polymers, 2 biopolymers, and 3 ceramics, ensuring diversity across performance metrics (thermal stability: 50–2400 °C; tensile strength: 7–250 MPa) while maintaining industrial feasibility for MD deployment.

**Figure 1:**
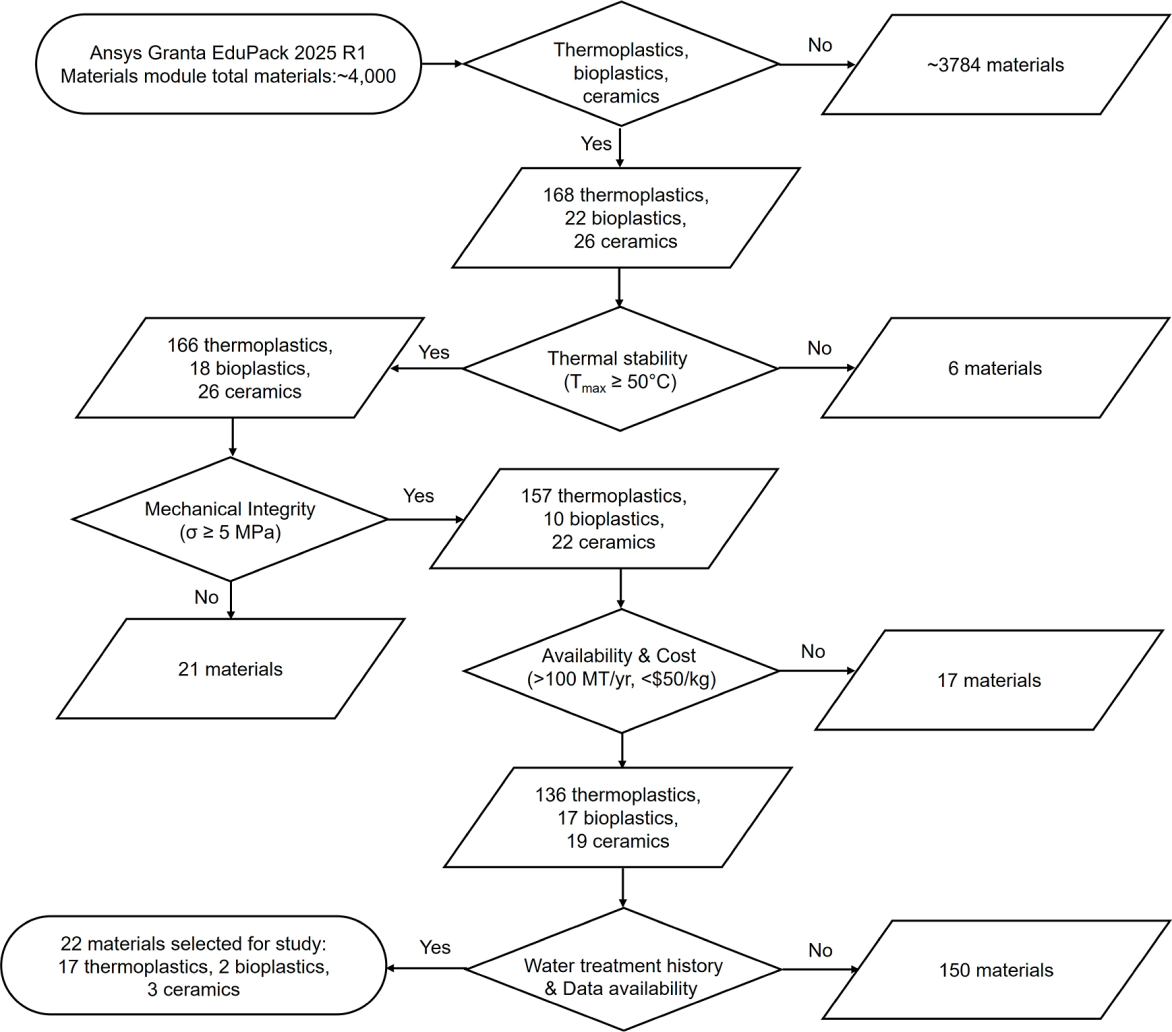
Hierarchical multistage material screening workflow for membrane fabrication candidates using Ansys Granta EduPack 2025 R1.

### 2.1 Chemical Compatibility Assessment

A compatibility assessment across diverse feed chemistries was conducted leveraging a chemical resistance database, which provided quantitative and categorical ratings as excellent, good, fair, and poor for material interactions with multiple chemical species based on standardized immersion testing protocols and long-term exposure studies. Materials were systematically evaluated for resistance to (i) organic solvents spanning aliphatic (hexane, heptane), aromatic (toluene, xylene), and polar aprotic (acetone, DMF) classes; (ii) strong acidic conditions encompassing pH < 2 environments with diverse inorganic (HCl, H_2_SO_4_, HNO_3_) and organic (acetic, citric) acid species; (iii) strongly alkaline conditions with pH > 12 containing sodium hydroxide, potassium hydroxide, and ammonium hydroxide solutions; (iv) food-grade process streams containing fats, proteins, and plant-derived organics representative of dairy, juice, and beverage processing; and (v) pharmaceutical-grade specifications requiring biocompatibility (ISO 10993), minimal extractables/leachables (USP Class VI), and sterility compatibility. Materials demonstrating “excellent” or “good” ratings across all chemicals within a category were classified as suitable; materials exhibiting “fair” or “poor” responses to any chemical species within the category were classified as incompatible. This multidimensional chemical compatibility assessment enabled material stratification into categories reflecting suitability for distinct application contexts, ranging from standard desalination to specialized resource recovery from aggressive industrial waste streams.

### 2.2 Life Cycle Environmental Assessment

Embodied energy and cradle-to-gate greenhouse gas emissions for each candidate material were quantified through the Ecoaudit module utilizing industry-standard life cycle inventory databases (USDA BioPreferred, ecoinvent v. 3.8, NREL U.S. Life Cycle Inventory). For each material, the baseline per-unit-mass environmental burden was established, encompassing raw material extraction, intermediate processing, polymer synthesis or ceramic sintering, and transportation to an industrial facility that assumed a 100 mile average distribution distance using a 3.5-ton truck. These production-phase metrics were subsequently scaled to a representative industrial-scale MD facility with a nominal water treatment capacity of 10,000 m^3^·day^–1^ and assuming a low seven-day membrane operational lifespan requiring membrane material of mass 1150 lb, establishing annual environmental demand associated with continuous membrane replacement cycles required for uninterrupted facility operation. End-of-life recovery potential was assessed through integrated analysis of (i) recycling efficiency and recovery rates from published literature and industry databases, with materials assigned recovery percentages reflecting current commercial recycling infrastructure maturity; (ii) energy recovered through thermal valorization or material recycling processes quantified using Ecoaudit end-of-life analysis; and (iii) associated greenhouse gas emission offsets achievable through recovered energy displacing fossil energy sources and recycled material displacing virgin material production. Net life cycle impact was calculated as production-phase burden minus end-of-life recovery benefit, enabling the identification of materials where robust recycling pathways substantially mitigate production-phase environmental costs. It is recognized that functional properties of membrane materials such as water vapor permeability and tensile strength may deviate from bulk polymer values due to membrane fabrication techniques including phase inversion, stretching, and electrospinning, which alter the microstructure, porosity, crystallinity, and effective thickness. Consequently, database-derived bulk properties in this study were employed for systematic screening and relative benchmarking to identify promising material candidates, with comparative experimental validation performed on three strategically selected commercial membranes (PP, PVDF, and PTFE) spanning the performance spectrum to account for processing-dependent property changes relevant to real-world membrane performance.

### 2.3 Experimental Validation

Commercially available PVDF, PTFE, and PP polymers were chosen to span the performance spectrum identified through informatics screening, representing moderate (PP), good (PVDF), and excellent (PTFE) intrinsic hydrophobicity and vapor transport characteristics based on database-derived water vapor transmission rates and contact angle properties. MD experiments were conducted by using a custom-designed module with an active membrane area of 4 cm^2^, enabling controlled thermal separation between hot feed (70 °C) and cold permeate (20 °C) streams. Operating conditions were selected to replicate typical industrial MD operation while maintaining controlled laboratory conditions for accurate performance assessment. Full experimental setup details, operational protocols, and process conditions are provided in Section S4 of Supporting Information. Membrane performance was assessed using the water vapor flux as the principal metric. Water vapor flux was calculated from permeate mass change according to

1 $$J=\frac{\Delta m}{A\cdot \Delta t}$$

where Δ*m* represents the mass of permeate collected (kg), *A* is the effective membrane area (m^2^), and Δ*t* is the operation time (h). The feed solution consisted of 35 g·L^–1^ NaCl to simulate seawater desalination conditions, with experiments conducted over 36 h operational periods to assess steady-state performance and temporal stability. Experimental results were directly compared against water vapor transmission-based theoretical predictions, enabling critical assessment of the informatics-driven framework’s predictive accuracy and its capacity to inform high-performance membrane selection for MD applications. Specifically, measured water vapor flux values were benchmarked against theoretical flux projections calculated from database-derived vapor transmission rates to validate the reliability of bulk property data for membrane performance prediction. The substantial differences in membrane thickness, porosity, and pore size among the three commercial membranes listed in Table 1 reflect their distinct fabrication methodologies: PTFE membranes are typically produced by paste extrusion and biaxial stretching, yielding highly porous node-fibril microstructures; PVDF membranes are commonly fabricated via phase inversion, producing interconnected sponge-like pore networks; and PP membranes are manufactured through melt-stretching (Celgard-type) processes, creating slit-like pore geometries. These morphological differences directly influence the relationship between intrinsic material vapor permeability and the observed membrane flux, as discussed in Section 3.4.1. While most of the properties of commercial membranes used for MD were obtained from the manufacturer’s data sheet, properties like porosity, contact angle, and liquid entry pressure (LEP) were measured using the gravimetric method, a Theta Lite optical tensiometer,^3^ and a custom dead-end filtration setup, respectively. The framework prioritizes a systematic material screening methodology; LEP and contact angle characterization represent important targets for future validation studies. The measured values are reported as follows.

**Table 1:** Characterization Properties of Commercial Membranes Used for DCMD Experimental Validation

membrane material	manufacturer	pore size (μm)	thickness (μm)	porosity (%)	contact angle (°)	liquid entry pressure (kPa)
PP	Tisch Scientific	0.45	200	60 ± 5	105 ± 3	320 ± 10
PVDF	Tisch Scientific	0.45	200	62 ± 2	96 ± 4	305 ± 15
PTFE	Tisch Scientific	0.45	200	77 ± 3	123 ± 7	335 ± 5

## 3 Results and Discussion

Systematic evaluation of twenty-two candidate membrane materials through Ansys Granta materials database assessment yielded comprehensive characterization of thermal, mechanical, transport, and chemical properties governing MD performance. Multidimensional material stratification emerged across all evaluated property categories, revealing distinct performance clustering that reflects fundamental differences in the material chemistry and fabrication potential. PP, PVDF, and PTFE materials were subjected to experimental validation through direct contact with MD testing. Concordance between Ansys Granta-predicted material property characteristics and predicted membrane performance confirmed the utility of materials informatics database approaches for membrane material discovery and selection.

### 3.1 Thermal Stability, Mechanical Integrity, and Vapor Transport as Key Performance Determinants

Materials selection boundaries were established through property trade-off analysis, following Ashby methodology principles. In the context of MD, thermal stability, mechanical strength, and vapor transport characteristics emerge as the primary material properties governing MD operational performance and long-term reliability. Thermal stability,^21^ quantified as maximum service temperature, and thermal conductivity^22^ establish the operational envelope for membrane deployment and directly determine the achievable temperature gradient driving the MD process.^23^ Tensile strength governs membrane mechanical integrity under thermal stresses and pressure differentials, while water vapor transmission rate determines permeate flux and process productivity.^24^ Analysis of maximum service temperatures across the evaluated material portfolio revealed distinct thermal capability stratifications, as shown in Figure 2. Ceramic materials, including zirconia, titania, and alumina, demonstrate maximum service temperatures exceeding 1000 °C, substantially surpassing the thermal requirements of conventional MD applications. This exceptional thermal stability positioned ceramics as optimal candidates for specialized industrial processes requiring elevated feed temperatures,^10^ including petrochemical refining and high-temperature reactor operations where feed conditions may reach or exceed 400 °C.^25^ Standard thermoplastic polymers including PE, PET, PP, PVDF, and PTFE exhibited maximum service temperatures between 100 and 250 °C, providing thermal compatibility with conventional MD feed temperatures typically ranging from 50 to 80 °C, while lower-performance polymers and biopolymers, including PS, ABS, styrene–butadiene–styrene (SBS), PVC, and PLA, exhibited maximum service temperatures approaching or below 100 °C. These materials demonstrated that the thermal stability is adequate only for operation near the lower thermal boundaries of MD processes, typically at feed temperatures near 50 °C, or these materials require reinforcement to withstand higher temperatures. Operation above these thermal thresholds risks membrane degradation, dimensional instability, and accelerated loss of separation efficiency, particularly for PVC and PLA. Consequently, material selection must be carefully aligned with anticipated operating temperatures to prevent premature membrane failure and maintain requisite salt rejection performance throughout the MD operational lifecycle.

**Figure 2:**
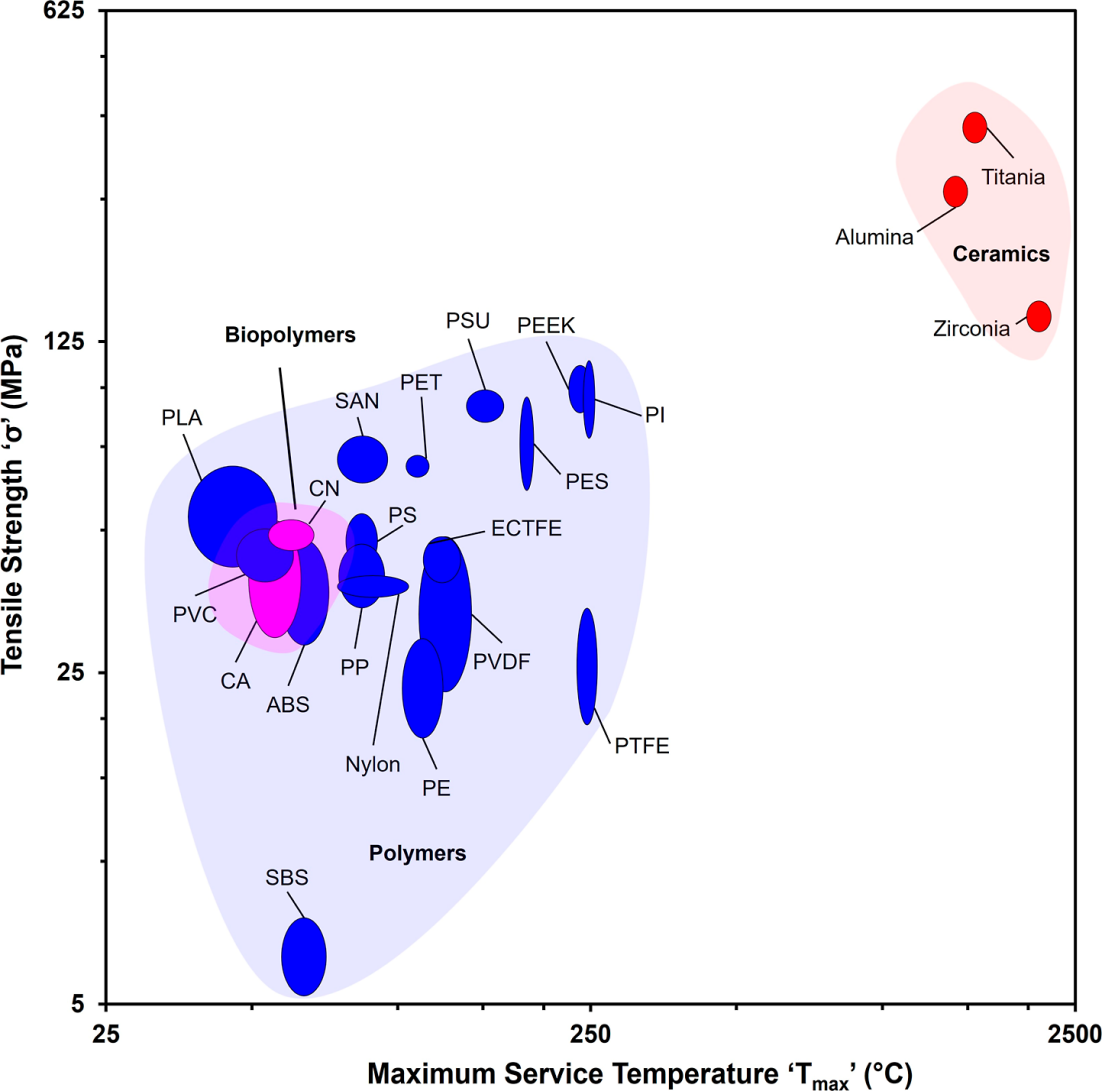
Ashby chart showing maximum service temperatures of candidate membrane materials from Ansys Granta, highlighting the thermal capability hierarchy from ceramics (>1000 °C) through engineering polymers (100–250 °C) to biopolymers and lower-performance polymers (<100 °C), and tensile strength distribution of candidate membrane materials from the Ansys Granta database, illustrating mechanical property stratification across ceramic, polymeric, and bioplastic material classes, with ceramics (titania, alumina, and zirconia) exhibiting exceptional strength (>200 MPa) compared to conventional polymers (30–120 MPa) and biopolymers (>40 MPa).

Tensile strength represents a critical material property governing membrane mechanical integrity directly influences the liquid entry pressure (LEP) threshold, which is the applied pressure at which nonvolatile feed components penetrate the membrane pores, compromising separation efficiency.^23^ Membrane materials with sufficient tensile strength resist deformation and wetting under the vapor pressure differentials generated across the hydrophobic membrane during operation, thereby maintaining a stable separation performance and preventing process failure. Analysis of tensile strength values extracted from Ansys Granta revealed substantial variation across the material portfolio shown in Figure 2.

An analogous three-tier stratification emerges for the mechanical properties. The 22 candidate materials exhibited clear mechanical stratification into three tiers: (i) ceramics (zirconia, titania, and alumina) demonstrating exceptional tensile strengths of 200–400 MPa, conferring superior mechanical resilience and capacity to sustain high circulation rates and pressure differentials without membrane degradation; (ii) engineering and commodity thermoplastics (PET, PP, PVDF, PTFE, PEEK, and ECTFE) spanning 30–120 MPa, providing adequate structural robustness for conventional MD operation against vapor pressure differentials and thermal stresses, with bioplastics (CN and nylon) similarly exhibiting strengths exceeding 40 MPa within this tier; and (iii) elastomeric and lower-performance polymers, with SBS exhibiting anomalously low strength below 10 MPa, rendering it unsuitable for direct MD deployment without reinforcement strategies such as supportive backing layers, electrospun nanofiber matrices, or increased membrane thickness beyond 100 μm.^21,23^ For materials in the lowest tier, mechanical limitations do not preclude MD application entirely, as multiple engineering strategies can offset these deficiencies, whereas ceramics in the highest tier confer intrinsic structural advantages that enable operation at higher feed flow velocities and elevated transmembrane pressures without additional reinforcement.^26–28^

Membrane selectivity in MD fundamentally depends on the capacity of the hydrophobic membrane to preferentially transport water vapor while effectively retaining nonvolatile species including dissolved salts, surfactants, organic contaminants, and macromolecular solutes.^29^ This selective transport mechanism relies on the interplay between membrane pore size distribution, which functions as a molecular sieve to exclude nonvolatile constituents, and vapor permeability,^30^ which subsequently governs the rate of water molecule transport across the membrane. The development of membranes exhibiting high rejection of nonvolatile species through reduced pore size typically compromises vapor transport kinetics, creating a fundamental trade-off between separation selectivity and process productivity. Achieving high water vapor transmission rates while maintaining adequate nonvolatile retention requires careful material selection and membrane fabrication strategies that optimize both transport properties and interfacial characteristics. Analysis of water vapor transmission rates across the evaluated material portfolio, as reported in Ansys Granta, reveals substantial heterogeneity in the intrinsic vapor permeability seen in Figure 3.

**Figure 3:**
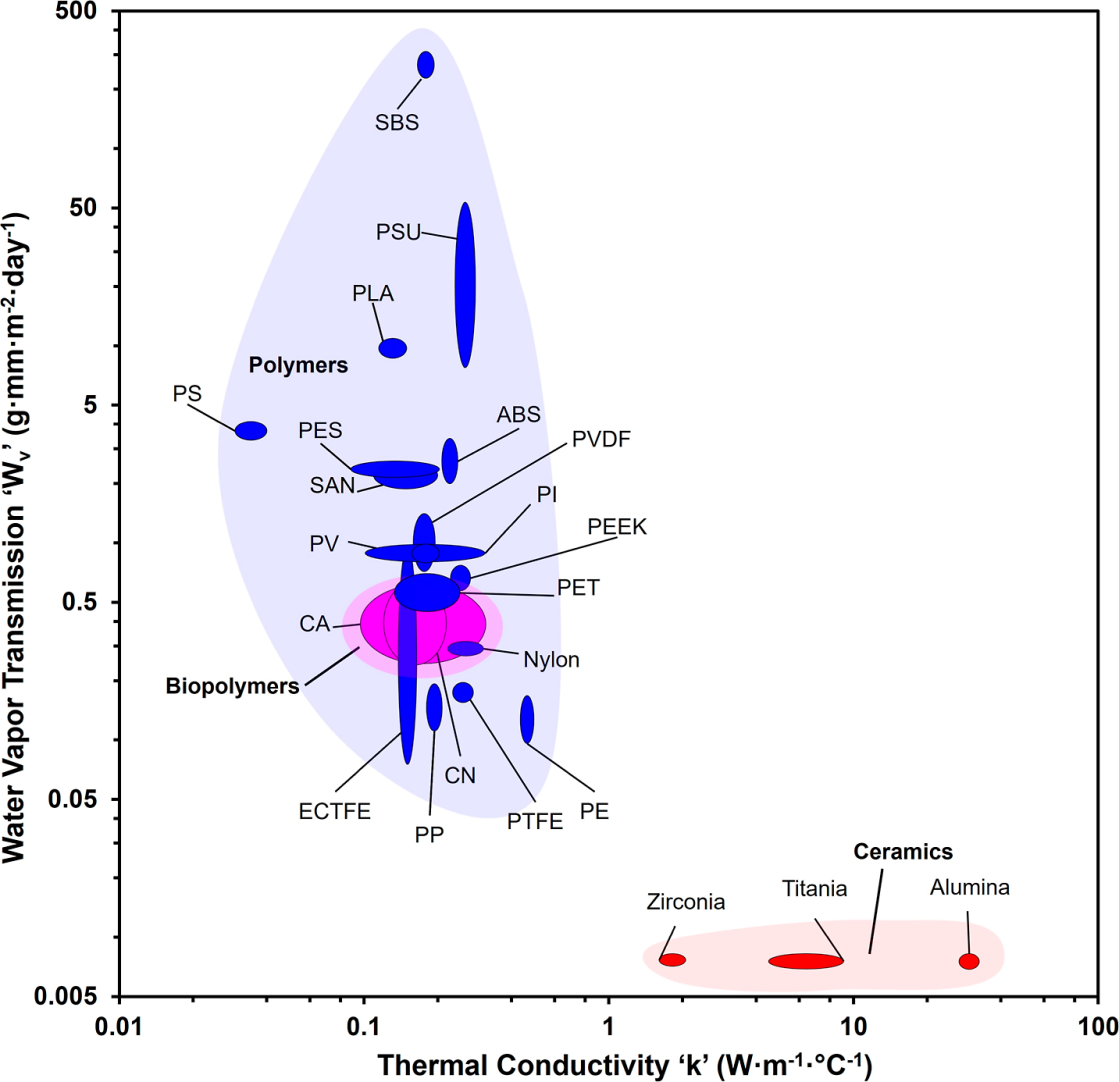
Water vapor transmission rate distribution of candidate membrane materials from the Ansys Granta database, illustrating the marked variation in intrinsic vapor permeability across material classes, with SBS and PSU demonstrating superior transmission rates (>30 g·mm·m^–2^·day^–1^), conventional polymers exhibiting moderate to high transmission (1–40 g·mm·m^–2^·day^–1^), and ceramics demonstrating substantially lower transmission rates (<0.1 g·mm·m^–2^·day^–1^), and thermal conductivity distribution of candidate membrane materials from the Ansys Granta database, illustrating thermal conductivity stratification across ceramic, polymeric, and bioplastic material classes, with ceramics (titania, alumina, and zirconia) exhibiting high thermal conductivity (>1 W·m^–1^.°C^1–^) compared to conventional polymers and biopolymers (0.03–0.3 W·m^–1^·°C^1–^).

The 22 candidate materials exhibited a four-order-of-magnitude span in intrinsic water vapor transmission, stratifying into three functionally distinct groups: (i) high-permeability elastomers and engineering polymers led by SBS (∼310 g·mm·m^–2^·day^–1^) and PSU (∼21 g·mm·m^–2^·day^–1^), with PES, PVC, SAN, ABS, PVDF, PLA, PI, and PS exceeding 1 g·mm·m^–2^·day^–1^ possessing sufficient intrinsic vapor permeability to enable compact pore architectures that simultaneously achieve high flux and effective nonvolatile rejection; (ii) moderate-permeability bioplastics (most formulations ≤0.5 g·mm·m^–2^·day^–1^), for which optimization of pore architecture, porosity, thickness, and hydrophobic surface treatment becomes critical to compensate for lower intrinsic permeability and balance competing demands of vapor transport and separation selectivity; and (iii) ceramics (titania, alumina, and zirconia) exhibiting intrinsic transmission rates below 0.01 g·mm·m^–2^·day^–1^, necessitating compensatory fabrication strategies, specifically ultrahigh porosity and minimal wall thickness, to enhance vapor diffusion pathways and reduce mass transfer resistance, though such architectural modifications risk compromising the mechanical advantages ceramics otherwise provide. This vapor transport hierarchy contrasts directly with the thermal and mechanical hierarchy, where ceramics rank highest: no single material class dominates across all three properties simultaneously, reinforcing the necessity of the multicriteria framework for application-specific material selection. For such materials, optimization of membrane fabrication parameters becomes critical to balance the competing demands of vapor transport and separation selectivity. Careful manipulation of pore architecture, porosity, thickness, and hydrophobic surface treatment can compensate for moderate intrinsic vapor permeability, enabling satisfactory performance across both mass transport and rejection metrics. In contrast, ceramic materials, such as titania, alumina, and zirconia, posed a unique challenge within the material selection framework. Despite exceptional thermal stability exceeding 1000 °C and extraordinary tensile strength exceeding 200 MPa, these materials exhibit remarkably low water vapor transmission rates, below 0.01 g·mm·m^–2^·day^–1^. This intrinsic limitation in vapor permeability necessitates compensatory fabrication strategies, specifically the development of ceramic membranes with extremely high porosity and minimal wall thickness, to enhance vapor diffusion pathways and reduce mass transfer resistance. While such architectural modifications can partially overcome inherent transport limitations, they may compromise the mechanical advantages conferred by ceramic materials, particularly when membranes are fabricated with porosities approaching theoretical limits. SBS exhibits an anomalously high water vapor transmission rate of 310 g·mm·m^–2^·day^–1^ attributable to its rubbery polybutadiene midblock, which provides high free volume and chain mobility that facilitate vapor transport far exceeding glassy or semicrystalline polymers such as PVDF, PTFE, and PP.

Another critical transport property that dictates the MD process is heat transfer; the membrane separates hot feed from cold feed, resulting in a vapor pressure difference. The vapor pressure difference is the major driving force for vapor transport across the membrane; the membrane material plays a significant role in ensuring no conductive heat loss from the hot side to the cold side. Membrane materials with low thermal conductivity are highly preferred to prevent energy leaks and act as an insulator. Membrane materials with high thermal conductivity will act as a conductor, resulting in significant heat loss and lowered vapor pressure difference. Out of 22 candidate membrane materials, PS has the lowest thermal conductivity, around 0.03 W·m^–1^·°C^1–^, which is extremely favorable for energy efficiency. All the other polymers and biopolymers have low thermal conductivity in the range of 0.1 and 1 W·m^–1^·°C^1–^, making them suitable for MD, while ceramics have higher thermal conductivity above 2 W·m^–1^·°C^1–^ with alumina exhibiting the highest thermal conductivity around 30 W·m^–1^·°C^1–^. The thickness of membranes must be optimized for materials with high thermal conductivity to ensure a thermal gradient across the hot feed and cold permeate is maintained.

### 3.2 Membrane Material Assessment for Critical Environments

A defining advantage of MD technology lies in its capacity to treat feed streams of highly variable and chemically aggressive composition. This versatility emerges directly from the chemical stability and selective permeability properties of membrane materials employed in MD systems. Evaluating membrane suitability for diverse applications requires chemical compatibility assessment across five feed-stream classes. These are saline water, organic solvents, strong alkalis (pH > 12), strong acids (pH < 3), and food-grade or medical-grade process streams. Such compatibility assessments guide material selection to prevent operational failures caused by membrane degradation, swelling, or dissolution when exposed to incompatible feed chemistries.

Material compatibility across diverse feed chemistries represents a critical determinant for successful MD deployment. Candidate materials were systematically classified for operational stability in high-salinity aqueous feeds and the organic solvent environments shown in Figure 4. Classifications draw on the Ansys Granta chemical resistance database and are cross-validated by the DCMD desalination trials conducted in this study. Chemical stability in saline aqueous environments is paramount for MD desalination, where feed streams may contain elevated concentrations of dissolved salts, trace organics, and suspended particulates. As shown in Figure 4, the majority of evaluated materials demonstrated excellent compatibility with saltwater, a finding corroborated through experimental DCMD performance testing on representative materials including PP, PVDF, and PTFE. All materials except SAN, PLA, and PSU were classified as excellent for saltwater applications. These materials can maintain dimensional stability, mechanical integrity, and vapor–liquid separation selectivity throughout extended exposure to concentrated brine solutions. Experimental validation through DCMD desalination testing confirmed database predictions for key materials. PTFE membranes achieved a consistent water vapor flux of ∼29 kg·m^–2^·h^–1^ and salt rejection >99% over 36 h operational periods with 35 g/L NaCl feed at 70 °C hot-side temperature. PP membranes, representing a cost-effective commodity polymer option, similarly demonstrated robust performance at ∼14 kg·m^–2^·h^–1^flux, with salt rejection >99%. PVDF membranes delivered a moderate flux of ∼11 kg·m^–2^·h^–1^ with salt rejection >99%, validating their suitability for standard desalination applications where extreme thermal or chemical resistance is not required. However, a limited subset of materials fell into the acceptable category for saltwater applications, including SAN and PLA. These materials may exhibit adequate short-term stability, but minor hydrophilicity shifts may result in flux decline during extended operation, suggesting the potential for gradual performance degradation under continuous high-salinity exposure. Notably, no materials evaluated in this study were classified as limited use or unacceptable for saltwater compatibility. The exceptional saltwater compatibility observed across nearly all material classes reflects the fundamental chemical stability of hydrophobic polymers and ceramics toward ionic species in aqueous environments. Salt ions remain fully solvated in the aqueous phase and do not penetrate hydrophobic membrane structures or induce polymer swelling, dissolution, or a chemical reaction. This near-universal saltwater compatibility provides substantial flexibility for the desalination material selection. Optimization can therefore focus on secondary criteria, including thermal stability, mechanical strength, vapor permeability, cost, and sustainability, rather than being constrained by aqueous chemical compatibility.

**Figure 4:**
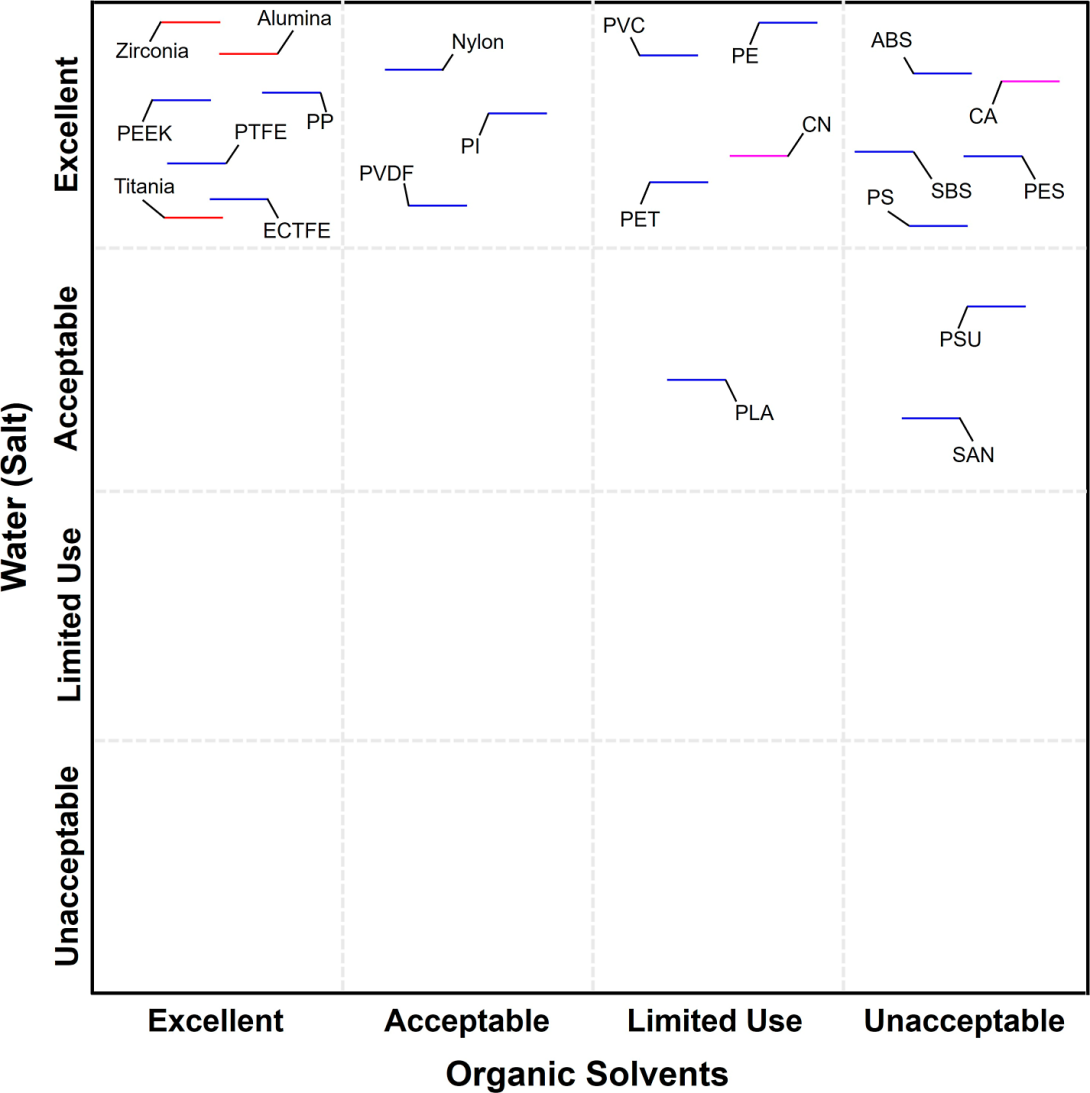
Chemical stability classification for desalination and organic solvent applications based on the Ansys Granta database and experimental DCMD validation. Nearly all materials (polymers, biopolymers, and ceramics) demonstrate excellent saltwater compatibility. Organic solvent resistance stratified into excellent (ceramics, PP, PTFE, ECTFE, PEEK), acceptable (nylon, PVDF, PI), limited use (PVC, PE, PET, PLA, CN), and unacceptable (SAN, PS, ABS, SBS, CA, PSU, PES) categories. Universal materials (ceramics, PTFE, PEEK) maintain stability across both environments; commodity polymers exhibit environment-specific limitations.

In contrast to the universally favorable saltwater compatibility, organic solvent resistance exhibited substantial material-dependent variation, as shown in Figure 4. Materials designated as excellent for organic solvent resistance included zirconia, titania, alumina, PP, PTFE, ECTFE, and PEEK. These materials exhibited comprehensive chemical inertness across diverse solvent classes including aliphatic hydrocarbons, aromatic compounds, chlorinated solvents, ketones, esters, and polar aprotic solvents. The superior chemical stability of these materials rendered them optimal candidates for robust industrial solvent processing environments, enabling broad applicability in organic wastewater treatment, solvent recycling operations, pharmaceutical purification, and catalyst recovery applications. Materials classified as acceptable for organic solvent applications including nylon, PI, and PVDF are resistant to a significant subset of common organic solvents but exhibit incompatibility with specific aggressive solvent classes. PVDF, while outstanding in aqueous desalination applications as validated experimentally in this study, showed susceptibility to strong polar aprotic solvents and certain aromatic compounds, necessitating feed-specific compatibility verification prior to deployment in organic-rich environments. This divergence between aqueous and organic solvent performance highlights the importance of comprehensive multienvironment compatibility assessment rather than extrapolating stability from single-feed chemistry evaluations. The limited use category encompassed materials showing significant susceptibility to dissolution or swelling when exposed to many common organic solvents, including PVC, PE, PET, PLA, and CN. While these materials performed excellently in aqueous salt solutions, their application in organic-rich feeds is restricted to tightly controlled compositions where exposure to aggressive solvents is rigorously minimized. Materials designated unacceptable for organic solvent exposure include SAN, PS, ABS, SBS, CA, PSU, and PES. Thus, the divergent compatibility profiles observed between saltwater and organic solvent environments reveal critical insights for membrane material selection. Nearly all evaluated materials demonstrated excellent resistance to saline aqueous feeds, whereas organic solvent compatibility stratified materials into clearly differentiated performance categories. This asymmetry reflects fundamental differences in polymer–solvent interaction mechanisms: ionic species in aqueous solution remain fully solvated and do not penetrate hydrophobic polymer matrices, whereas organic solvents can induce polymer swelling through favorable enthalpic interactions or complete dissolution when solubility parameters align.

Additionally, membrane stability under extreme pH conditions represents a critical performance criterion for industrial applications involving caustic or acidic waste streams. Figure 5 presents a systematic compatibility assessment of 22 candidate membrane materials under exposure to strong alkalis at pH > 12 and strong acids at pH < 3. Highly alkaline waste streams are ubiquitous across diverse industrial sectors. In pulp and paper manufacturing, alkaline pulping processes generate caustic effluents containing NaOH concentrations exceeding 100 g L^–1^ at pH > 13. Textile processing and dyeing operations employ strong alkaline solutions for mercerization, scouring, and dye fixation, producing waste streams with pH 11–14. Metal finishing and electroplating industries utilize alkaline cleaning baths and etching solutions, while petroleum refining generates caustic wash streams during desulfurization and hydrocarbon processing. Battery manufacturing including both lithium-ion and alkaline produces highly caustic electrolyte waste, and soap and detergent production involves saponification reactions in concentrated alkali media. Semiconductor fabrication employs alkaline photoresist developers and chemical-mechanical planarization slurries, and aluminum anodizing operations generate alkaline cleaning and etching effluents. As demonstrated in Figure 5, materials exhibiting excellent resistance to strong alkalis include PVC, PE, SAN, ABS, PP, high-performance fluoropolymers PTFE, ECTFE, and PEEK and alumina membranes. These materials demonstrate no chemical degradation, dimensional instability, or mechanical property deterioration under prolonged exposure to concentrated caustic solutions, maintaining structural integrity essential for reliable MD operation. Materials classified as acceptable for alkaline environments include SBS, PS, PSU, and zirconia. These materials withstand most alkaline conditions but may exhibit minor surface etching or gradual property changes under extended exposure to highly concentrated caustic solutions or elevated-temperature alkaline environments, necessitating periodic performance monitoring during operation. The limited use category encompasses PET, CN, nylon, PVDF, PES, and titania, all of which demonstrate susceptibility to alkaline hydrolysis or surface degradation mechanisms. PET and polyamides undergo ester and amide bond cleavage, respectively, under alkaline conditions, while PVDF exhibits gradual dehydrofluorination in strong caustic media. These materials require rigorous pH control and are restricted to applications where alkaline exposure is minimized or neutralized prior to membrane contact. Materials designated unacceptable for alkaline environments include PLA, CA, and PI, which undergo rapid hydrolytic degradation through ester bond cleavage in alkaline media. Bioplastic materials are fundamentally incompatible with caustic waste streams and are precluded from deployment in any application involving pH > 10.

**Figure 5:**
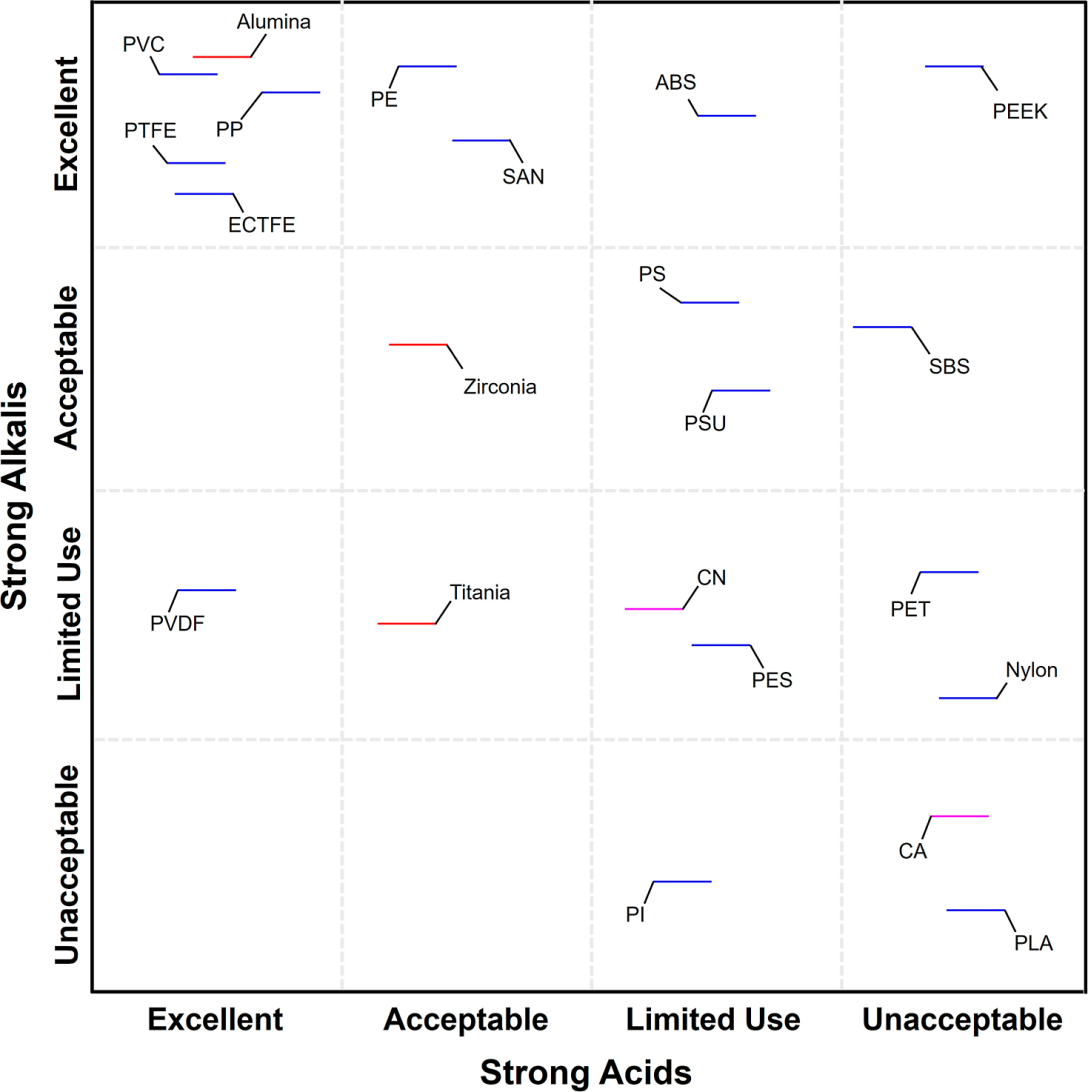
Four-tier compatibility assessment (excellent, acceptable, limited use, and unacceptable) for membrane materials exposed to strong alkalis (pH > 12) and strong acids (pH < 3). Fluoropolymers (PVDF, PTFE, ECTFE) and alumina demonstrate universal stability across both extreme pH environments; commodity polymers exhibit environment-specific limitations; PEEK shows excellent alkaline but poor acid resistance; PLA and CA undergo rapid degradation in both conditions. Classifications based on Ansys Granta materials database evaluation for industrial waste treatment applications including pulp and paper, metal processing, mining, pharmaceuticals, and semiconductor manufacturing.

Strongly acidic industrial waste streams are equally prevalent across the manufacturing sectors. Metal pickling and acid cleaning operations in steel production generate waste containing HCl, H_2_SO_4_, HNO_3_, and HF at pH < 1. Mining and mineral processing, particularly copper, zinc, and rare earth extraction, produce acid mine drainage and leaching solutions with pH 1–3. Pharmaceutical synthesis involves acid-catalyzed reactions and purification steps generating acidic mother liquors. Lead-acid and lithium-ion battery recycling produces sulfuric and hydrofluoric acid waste streams. Semiconductor fabrication employs concentrated acid cleaning such as HF, HCl, H_2_SO_4_, and etching solutions. Food and beverage processing generates acidic waste from citric acid production, carbonation operations, and fermentation processes. Petrochemical alkylation units utilize sulfuric or hydrofluoric acid catalysts, while titanium dioxide (TiO_2_) pigment production involves sulfuric acid digestion of ilmenite ore. Figure 5 reveals a noticeably different compatibility profile compared with alkaline environments. Materials demonstrating excellent acid resistance include high-performance fluoropolymers PVDF, PTFE, and ECTFE, commodity polymers PVC and PP, and alumina ceramics. The superior acid stability of fluoropolymers reflects the exceptional strength of C–F bonds resistant to protonation or hydrolysis, positioning these materials as optimal candidates for aggressive acidic waste treatment applications. Materials classified as acceptable for acid environments include PE, SAN, zirconia, and titania. These withstand most common mineral acids but may degrade under highly oxidizing conditions such as concentrated HNO_3_, hot H_2_SO_4_, and HF through surface attack or oxide dissolution. The limited use category encompasses PS, ABS, CN, PI, PES, and PSU. These materials show adequate stability at mild acidity (pH 3–5). Progressive degradation occurs under strong acidic conditions via aromatic ring sulfonation (PS, ABS), glycosidic bond hydrolysis (CN), or sulfone linkage cleavage (PSU). Materials designated unacceptable for acidic environments include PET, SBS, CA, PLA, nylon, and PEEK. PET, CA, and PLA undergo rapid acid-catalyzed ester hydrolysis, while polyamides experience amide bond cleavage. The most industrially consequential finding from the pH compatibility analysis is PEEK’s orthogonal pH profile: it achieves excellent resistance across alkaline environments (pH > 12), among the best of the 22 candidates, yet is classified as unacceptable for strongly acidic conditions (pH < 3) due to protonation and chain scission of its ether and ketone linkages. To the authors’ knowledge, this is the first systematic, database-driven quantification of PEEK’s pH asymmetry across a 22-material comparative set under standardized MD deployment criteria. This finding carries direct industrial safety implications: PEEK is frequently specified in high-performance process equipment, heat exchangers, and separation systems exposed to variable pH streams. A PEEK membrane deployed in a multistage process involving both caustic cleaning (NaOH, pH > 13) and acid pickling steps (HCl/H_2_SO_4_, pH < 1) common in metal finishing, semiconductor fabrication, and pharmaceutical synthesis would perform reliably under alkaline exposure but undergo rapid structural failure upon acid contact. This orthogonal behavior cannot be inferred from generalized chemical resistance ratings; it requires environment-specific, mechanism-level compatibility assessment of the type presented here.

The saltwater–organic solvent compatibility asymmetry identified in Section 3.2 carries direct implications for industrial deployment practice. Because nearly all 22 candidate materials are safe for aqueous desalination, only a subset—PVDF, PTFE, ECTFE, PP, and ceramics—maintains stability in organic-rich feeds, and the feed chemistry at the deployment site, not material cost or flux ranking, should serve as the primary filter in any material selection decision. This has a regulatory dimension as well: environmental permitting of MD systems treating mixed organic-aqueous industrial effluents such as pharmaceutical mother liquors, mining leachates, and semiconductor rinse waters increasingly require documentation of membrane chemical compatibility across the full feed composition range and not just performance under saline conditions. The Ansys Granta chemical resistance database employed here provides precisely this multienvironment compatibility profile at the screening stage, before any membrane is fabricated or tested, enabling regulatorily defensible material choices from the outset of system design. The specific material-by-material classifications presented in Figure 4, demonstrating excellent through unacceptable ratings, represent systematic evaluations derived from the Ansys Granta chemical resistance database utilized in the present study, cross-referenced against standardized immersion testing protocols. Only a limited subset of materials, specifically PVDF, PTFE, ECTFE, PVC, PP, and alumina, demonstrated excellent or acceptable performance across both alkaline and acidic environments, positioning these materials as universal candidates for industrial waste treatment applications where feed pH may vary unpredictably. Several materials exhibited strong environment-specific preferences: PEEK demonstrates excellent alkaline resistance but failed catastrophically under acidic conditions, while titania and zirconia show acceptable acid resistance but limited alkaline stability. For MD systems treating feeds with variable or unpredictable pH, material selection must begin with universal pH-stable candidates: PVDF, PTFE, ECTFE, and alumina. Applications with well-defined alkaline feeds may also draw from PE, PVC, ABS, PP, and PEEK, provided that upstream pH neutralization is confirmed. Biopolymers (PLA, CA) and polyamides must be excluded from any application involving pH extremes below 3 or above 10. Additional compatibility of these 22 materials with food and medical applications is reported in Figure S1 of Supporting Information.

### 3.3 Environmental Impact Assessment and Life Cycle Analysis

Quantification of environmental impacts associated with membrane material production and end-of-life recovery represents an essential dimension of comprehensive material selection, particularly for industrial-scale deployment where cumulative resource consumption and carbon footprint become economically and environmentally significant. This analysis integrates an Ansys Granta Ecoaudit sustainability assessment tool to evaluate the embodied energy and greenhouse gas emissions associated with fabricating sufficient membrane material to sustain continuous operation of a representative large-scale desalination facility. Analysis was conducted on the basis of a nominal 10,000 m^3^·day^–1^ seawater desalination facility operating with a seven-day membrane lifespan, requiring continuous membrane replacement cycles to maintain uninterrupted treatment capacity. Under these operational assumptions, the annual energy requirement and associated CO_2_ emissions for membrane fabrication were quantified using Ansys Granta Ecoaudit for all evaluated material candidates summarized in Figure 6a,b. Results reveal pronounced differentiation in the environmental burden across material classes. Polymeric materials demonstrate substantially elevated energy demand, with PI, PEEK, PTFE, and PES emerging as the four most energy-intensive materials, each requiring annual fabrication energy exceeding 5 MJ·year^–1^. These high-performance engineering polymers necessitate computationally demanding synthesis processes and energy-intensive polymerization procedures, driving their elevated environmental footprints. In contrast, bioplastic and ceramic materials demonstrate markedly lower operational energy demands. CN, PVC, and ceramic materials, including alumina, titania, and zirconia, exhibit substantially reduced annual energy requirements, typically below 1 MJ·year^–1^. The remaining polymeric materials occupy intermediate positions, each consuming less than half the annual energy demanded by PI. Greenhouse gas emission profiles parallel energy demand patterns across the material portfolio. High-performance polymers such as PI, PEEK, PTFE, and PES generate the largest annual CO_2_ emissions, collectively accounting for disproportionate contributions to the carbon footprint of membrane production. Conversely, conventional polymers including PP, PE, PS, biopolymers (PLA), and ceramics (alumina) demonstrate substantially lower carbon emissions. This stratification enables material selection strategies that explicitly target environmental objectives; selection of lower-carbon-footprint materials provides a direct mechanism for achieving corporate sustainability targets and reducing operational life cycle carbon intensity.

**Figure 6:**
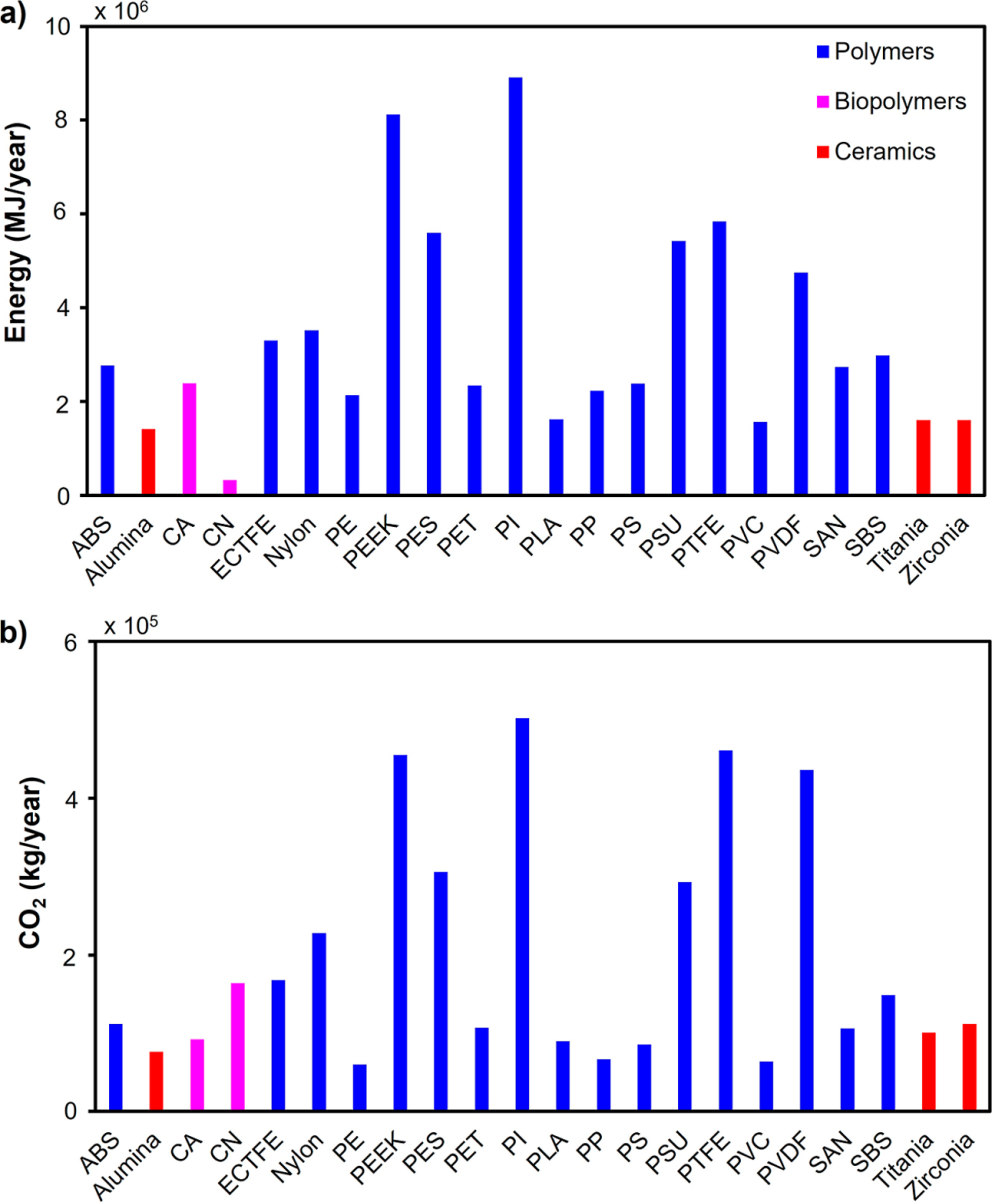
Annual environmental burden of candidate membrane materials during fabrication and replacement cycles for a 10,000 m^3^·day^–1^ desalination facility: (a) cumulative energy demand (MJ·year^–1^) showing high-performance polymers (PI, PEEK, PTFE, PES) as energy-intensive and ceramic materials as efficient alternatives; (b) associated greenhouse gas (CO_2_) emissions (kg·year^–1^) demonstrating similar stratification across material classes, with polymeric materials substantially exceeding ceramic alternatives.

Critical consideration of membrane material sustainability extends beyond operational production impacts to encompass end-of-life recovery potential, recycling efficiency, and associated energy and emission offsets in Table S2. Analysis of end-of-life scenarios reveals that PEEK membranes, despite high operational energy demand, achieve the highest energy recovery potential among high-performance polymers, recovering approximately 1.0 × 10^5^ MJ through recycling processes. This substantial recovery offset reflects PEEK’s compatibility with advanced recycling technologies and high-value secondary material recovery pathways. Among the four most energy-intensive materials, PES demonstrates the second-highest energy offset potential of 7.1 × 10^4^ MJ, followed by PI and PTFE. Notably, CN demonstrates anomalously high energy offset relative to its minimal operational energy demand, reflecting its compatibility with efficient biological and thermal decomposition pathways that yield energy recovery. Across the portfolio of lower-carbon-footprint materials, PS achieves the highest CO_2_ emission offset (9.3 × 10^2^ kg CO_2_), followed by PE, PP, and PLA. These materials exhibit established recycling infrastructure and secondary material markets that enable substantial emission reductions through recovery pathways. The integration of operational production impacts with end-of-life recovery potential reveals a more nuanced sustainability landscape than operational metrics alone suggest. While PI, PEEK, PTFE, and PES impose the highest operational environmental burdens, their participation in advanced recycling and recovery networks substantially mitigates lifecycle carbon intensity. Conversely, materials with minimal operational footprints may contribute minimal recovery value, presenting trade-offs between production-phase sustainability and end-of-life circularity. Selection of optimal materials, therefore, requires a holistic consideration of both production and recovery phases to align with specific sustainability objectives and facility infrastructure capabilities.

This study reports a lifecycle inversion finding with broad engineering significance: PEEK and PES, ranked first and second in production-phase energy demand among all 22 candidates, each exceeding 5 MJ·year^–1^ at the 10,000 m^3^·day^–1^ facility scale, achieving net lifecycle environmental benefit through established recycling infrastructure and recovering approximately 1.0 × 10^5^ MJ and 7.1 × 10^4^ MJ, respectively. This inversion fundamentally invalidates the widely applied heuristic that the production carbon footprint is a reliable proxy for material environmental performance. It directly challenges material specification practices across chemical process engineering, packaging, automotive lightweighting, and biomedical device sectors, where production-phase impact is routinely used as the primary sustainability criterion. For circular water economy contexts specifically, these findings mandate that LCA for membrane material selection must integrate full end-of-life recovery accounting; any sustainability ranking based on embodied energy alone will systematically misidentify the environmentally preferable choice.

### 3.4 Multidimensional Material Performance Analysis and Selection Optimization

#### 3.4.1 Water Vapor Flux Prediction

To enable rapid screening of membrane candidates without exhaustive experimental testing, an empirical correlation was developed, relating intrinsic material properties to anticipated MD performance. The water vapor flux prediction model incorporates material W_V_ alongside assumed membrane morphological parameters (porosity, tortuosity, thickness) and operational driving force (transmembrane vapor pressure differential):

2 $$J_{pred}=\alpha \left(\frac{\Delta p}{\delta }\right)\left(\frac{\varepsilon }{\tau }\right)\left[W_{v}+\beta \right]$$

where *J*_pred_ is the predicted vapor flux (kg·m^–2^·h^–1^), *W*_v_ is water vapor transmission (g·mm·m^–2^·day^–1^), ε is porosity (dimensionless), τ is tortuosity (dimensionless), δ is membrane thickness (μm), Δ*p* is vapor pressure difference (bar), and empirically fitted parameters α (bar^–1^) is 1775 and β (g·mm·m^–2^·day^–1^) is 20. Validation of Ansys Granta-derived material selection recommendations through direct experimental comparison represents a critical step in establishing the reliability and practical applicability of database-driven material assessment. MD performance testing under standardized conditions (hot feed 70 °C and cold permeate 20 °C) using the custom-designed setup shown in Figure S2 for three commercially available polymeric membranes PP, PVDF, and PTFE was conducted to evaluate whether Ansys Granta-based predictions aligned with observable performance metrics. The three evaluated membranes demonstrated successful desalination of 35 g·L^–1^ NaCl solutions without requiring postfabrication surface modification, reflecting the intrinsic hydrophobicity of these polymeric materials. PP membranes achieved a water vapor flux of 14 ± 2 kg·m^–2^·h^–1^, PVDF membranes produced 11 ± 3 kg·m^–2^·h^–1^, and PTFE membranes delivered 29 ± 4 kg·m^–2^·h^–1^. Predicted fluxes demonstrated strong agreement with experimental measurements with coefficient determination close to 1, as shown in Figure 7. This correspondence between the predicted membrane vapor flux and observed experimental flux validates the utility of Ansys Granta property data for prospective performance estimation under the same membrane structural characteristics and operating conditions.

**Figure 7:**
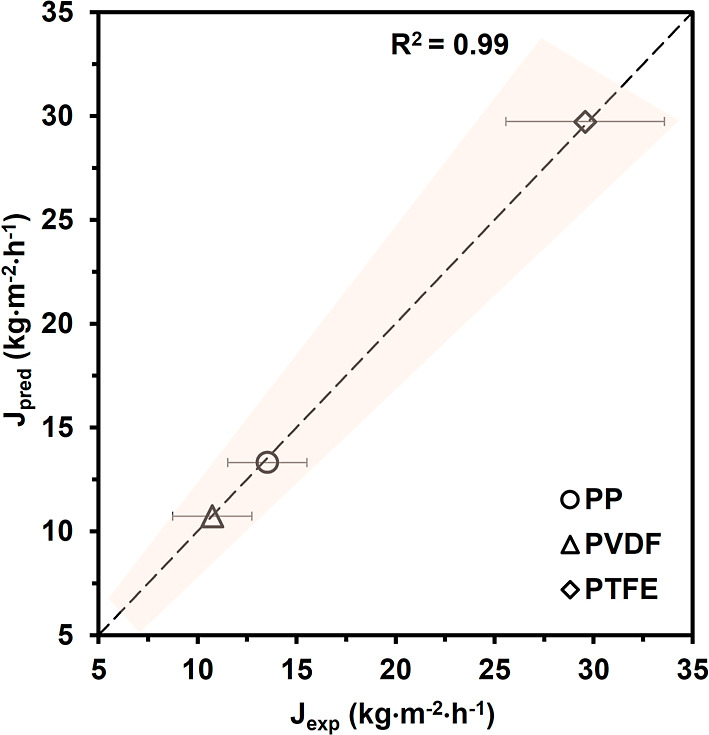
Parity plot comparing experimental and predicted values of vapor flux *J*; the dashed line represents the agreement between experimental and predicted vapor flux with a high coefficient of determination of 0.99. PTFE membranes exhibited the highest *J*_exp_ of 29 ± 4 kg·m^–2^·h^–1^ and J_pred_ of 29.7 kg·m^–2^·h^–1^, followed by PP membranes with *J*_exp_ of 14 ± 2 kg·m^–2^·h^–1^ and *J*_pred_ of 13.3 kg·m^–2^·h^–1^; PVDF membranes produced *J*_exp_ of 11 ± 3 kg·m^–2^·h^–1^ and *J*_pred_ of 10.7 kg·m^–2^·h^–1^.

The experimental validation presented in this study was designed as strategic verification of the materials informatics framework’s predictive utility rather than as an exhaustive characterization of commercial membrane performance. The selection of three commercially available membranes spanning distinct performance tiers of polypropylene representing moderate capability, polyvinylidene fluoride representing good performance, and polytetrafluoroethylene representing excellent thermal and chemical stability provides validation across the materials spectrum while maintaining experimental efficiency. The strong correlation (*R* ^2^ ∼ 1) between Ansys Granta-predicted vapor transmission rates and experimentally measured water vapor flux demonstrates that bulk material properties derived from databases reliably predict relative membrane performance under controlled laboratory conditions, thereby validating the informatics-driven screening approach for prospective material identification. The framework’s primary scientific contribution centers on systematic material selection methodology and multicriteria optimization, rather than on comprehensive membrane characterization or fouling behavior prediction. Future work warrants the extension of this methodology through validation with real industrial waste streams of variable chemistry, integration with alternative MD configurations, such as air gap MD, sweeping gas MD, and vacuum MD, and exploration of emerging materials, including metal–organic frameworks and mixed-matrix composites. This expansion will enable refinement of material rankings for application-specific contexts and further strengthen the foundation for circular water economy implementation across diverse industrial sectors.

Application of the predictive model eq 2 across all 22 candidate materials yielded anticipated flux values spanning 2 orders of magnitude as shown in Figure 8, from 24.3 kg·m^–2^·h^–1^ for low-W_v_ ceramics and fluoropolymers to 413.7 kg·m^–2^·h^–1^ for SBS, which is attributable to its exceptionally high intrinsic *W*_v_ of 310 g·mm·m^–2^·day^–1^. The SBS-predicted flux represents a theoretical upper-bound estimate derived from bulk material *W*_v_ data, assuming standard membrane morphological parameters. This value has not been experimentally validated for SBS membranes in MD configuration. Given SBS’s extremely low tensile strength at <10 MPa and known incompatibility with numerous organic solvents and strong acids discussed previously in Section 3.2, achieving the assumed porosity and thickness parameters in a structurally stable SBS membrane presents significant fabrication challenges. Experimental validation with SBS-based membranes is required before practical deployment considerations. High-performance polymers including PSU at 66.8 kg·m^–2^·h^–1^, PLA at 37.0 kg·m^–2^·h^–1^, and ABS at 28.3 kg·m^–2^·h^–1^ emerged as promising high-flux candidates, while materials with inherently low W_v_ ceramics, PTFE, ECTFE, and PEEK clustered in the 24–25 kg·m^–2^·h^–1^ range, requiring optimized membrane fabrication (ultrathin layers, high porosity) to achieve competitive permeate productivity. Comprehensive analysis of predicted MD performance reveals that membrane material selection, while strategically important, represents only one dimension of a complex multifactorial system, determining experimental outcomes. Membrane structural properties including thickness, porosity, and pore size distribution themselves, a function of selected fabrication methodology, substantially modulate transport properties and separation performance independent of bulk material selection. Interfacial characteristics including contact angle hysteresis, pore size distribution heterogeneity, and wetting behavior similarly exert a profound influence on MD flux and rejection characteristics.

**Figure 8:**
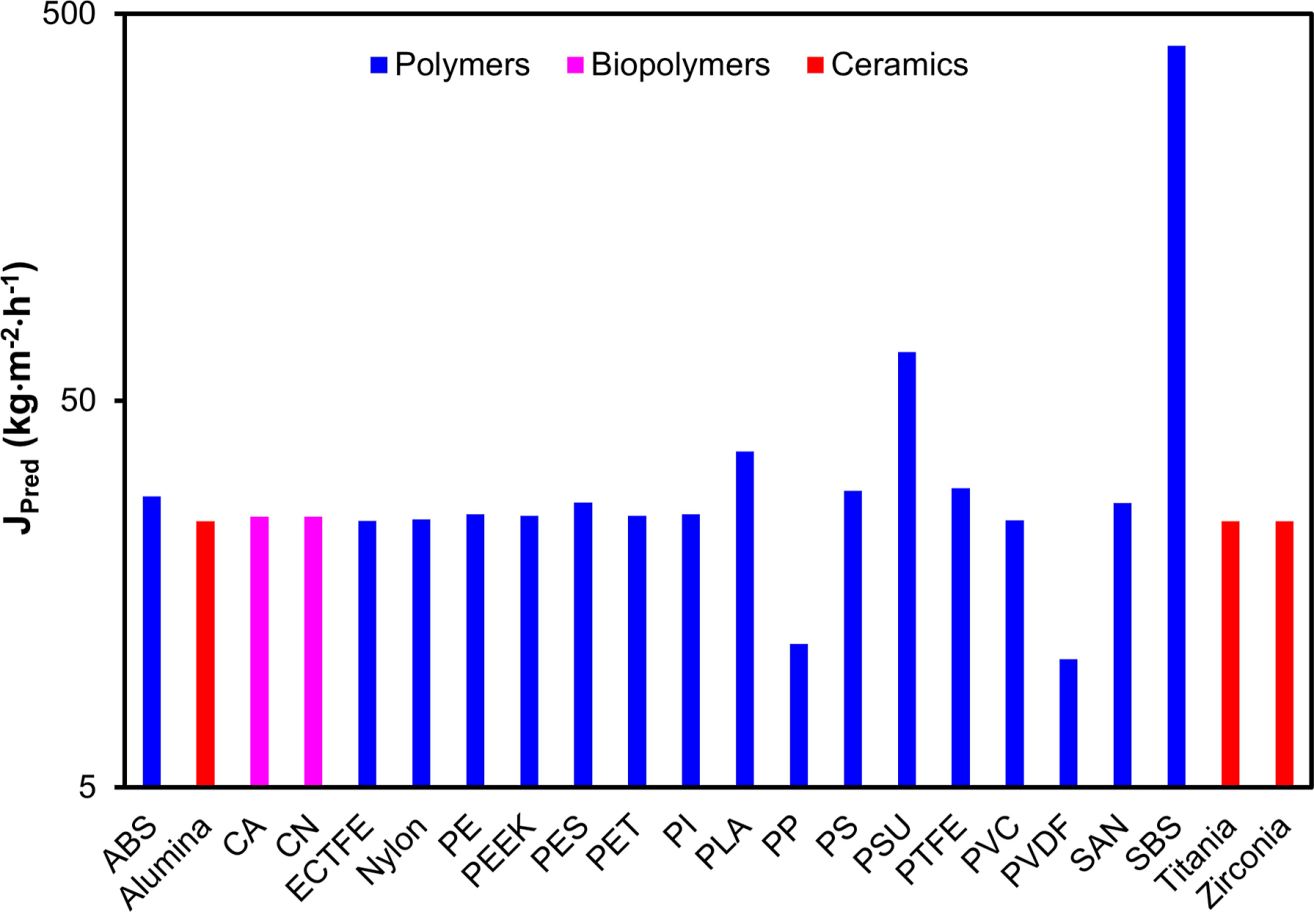
Predicted MD permeate flux for 22 membrane materials. Flux spans 2 orders of magnitude from 24.3 kg·m^–2^·h^–1^ for low-Wv ceramics/fluoropolymers: PTFE, ECTFE, and PEEK; alumina, titania, and zirconia to 413.7 kg·m^–2^·h^–1^ for SBS, consistent with its intrinsic W_v_ = 310 g·mm·m^–2^·h^–1^. Notable polymer predictions: PSU: 66.8 kg·m^–2^·h^–1^, PLA: 37.0 kg·m^–2^·h^–1^, and ABS: 28.3 kg·m^–2^·h^–1^. While eq 2 reflects bulk vapor permeability, experimental MD flux also depends on morphology such as thickness, porosity, pore-size distribution, and interfacial wetting behavior.

An important methodological consideration is the sensitivity of min–max normalization to outlier values. SBS’s water vapor transmission rate at 310 g·mm·m^–2^·day^–1^) exceeds the next highest polymer such as PSU, approximately 36 g·mm·m^–2^·day^–1^ by nearly an order of magnitude. This disparity compresses the normalized *W*_v_ scores of all other materials toward zero, effectively diminishing the flux contribution to their composite Π values. Consequently, commonly deployed MD polymers such as PE (*W*_v_ ∼ 0.5), PVDF (∼8), and PP (∼1 g·mm·m^–2^·day^–1^) receive disproportionately low flux scores despite possessing adequate vapor transport properties for practical MD applications. To evaluate the robustness of the performance rankings, a sensitivity analysis was conducted by recalculating Π with SBS excluded from the normalization pool (Table S5). Under this adjusted normalization, the flux contributions of conventional polymers increase substantially: PSU emerges as the top-ranked material under flux-priority criteria, while PVDF and PES improve by 3–5 ranking positions under equal-importance weighting. Under sustainability-priority weighting, rankings remain largely unaffected because the Wv weight (0.1) is insufficient to significantly shift composite scores. Practitioners should consider both full-range and SBS-excluded rankings when making deployment decisions, recognizing that SBS faces significant practical barriers including low mechanical strength, limited chemical compatibility, and moderate thermal stability that the current Π formulation does not explicitly penalize. Future refinements could incorporate minimum threshold constraints, where materials failing critical requirements (e.g., tensile strength <10 MPa) receive penalty factors in the composite index.

While database-derived bulk material properties may not directly translate to absolute membrane performance due to processing-induced changes in porosity, pore size distribution, thickness, and crystallinity achieved through techniques such as phase inversion, stretching, and electrospinning, the materials informatics approach employed here successfully captures the relative performance ranking of candidate materials. Experimental validation confirmed that Ansys Granta predictions correlated with measured performance (*R* ^2^ ∼ 1) and correctly ranked the three commercial membranes (PTFE > PP > PVDF in water vapor flux), demonstrating the framework’s utility for comparative materials screening despite limitations in absolute flux prediction. The methodological separation between material property selection, the focus of this study, and fabrication process optimization represents an intentional scope boundary that enables systematic material evaluation without requiring detailed process simulations for each candidate material. This staged approach provides efficiency gains for initial screening while acknowledging that superior membrane performance ultimately requires integration with optimized fabrication routes. As morphological constraints specific to each material class become better characterized through expanded experimental data sets, the Π framework can be refined to incorporate material-specific porosity and thickness limitations, progressively enhancing predictive accuracy. This iterative improvement pathway positions materials informatics as a scalable platform for accelerating membrane technology development within circular water economy contexts.

#### 3.4.2 Cross-Property Correlation Assessment

To elucidate intrinsic structure–property relationships governing membrane performance, the Pearson correlation coefficient (*r*) with associated *p*-values denoting the probability of observing the correlation under the null hypothesis of no association was calculated between key material properties extracted from the Ansys Granta database. The correlation analysis encompassed six critical parameters: maximum service temperature (*T*_max_), tensile strength (σ), water vapor transmission (*W*_v_), thermal conductivity (*k*), embodied energy (*E*), and material cost (*C*_m_), evaluated across 22 candidate materials spanning polymeric, bioplastic, and ceramic classes. Since Pearson correlation is tested among 6 properties, 15 independent comparisons were made. To consider the increased likelihood of false positive outcomes when determining multiple pairwise correlations, the Bonferroni correlation was applied. With the assumption of nominal significance level α = 0.05, the corrected threshold for significance was obtained by dividing α by the number of independent comparisons (*m*), giving a stringent criterion and ensuring that only robust correlations are interpreted as meaningful.

3 $$\alpha_{adjusted}=\frac{\alpha }{m}$$

Based on α_adjusted_ = 0.00333, only two correlations survived the corrected significance threshold as shown in Figure 9: (i) maximum service temperature versus tensile strength (*r* = 0.77, *p* = 0.00003), indicating that materials capable of withstanding higher operational temperatures also have superior mechanical robustness, and (ii) tensile strength and thermal conductivity (*r* = 0.67, *p* = 0.00073), implying close interdependence between mechanical strength and phonon transport pathways arising from improved crystallinity and bonding strength. However, other correlations did not meet the stringent correlated significance threshold and should be interpreted with caution as potential rather than confirmed relationships. *T*_max_ also had moderate correlation with material cost C_m_ (*r* = 0.56, *p* = 0.00634), suggesting a trade-off between high-temperature performance and the material’s economic feasibility. Conversely, maximum service temperature demonstrated a weak negative correlation with embodied energy (*r* = −0.22), suggesting that thermally robust materials do not necessarily impose elevated production-phase environmental burdens, a critical insight for sustainable material selection. The water vapor flux W_v_ and embodied energy E have a weak correlation (|*r*| < 0.3) with other properties that highlight their independence from the intrinsic nature of the material. Instead, these two properties depend on surface-level or structural features like porosity, pore size distribution, tortuosity, etc., emphasizing its tuning via fabrication parameters rather than material chemistry alone. Together, these relationships reflect a coupled dependence on thermo-mechanical and transport properties, underscoring a key trade-off between performance and cost that should guide the material selection for the MD system. A detailed economic feasibility and cost-performance trade-off analysis for the materials in consideration is provided in Supporting Information Section S6.

**Figure 9:**
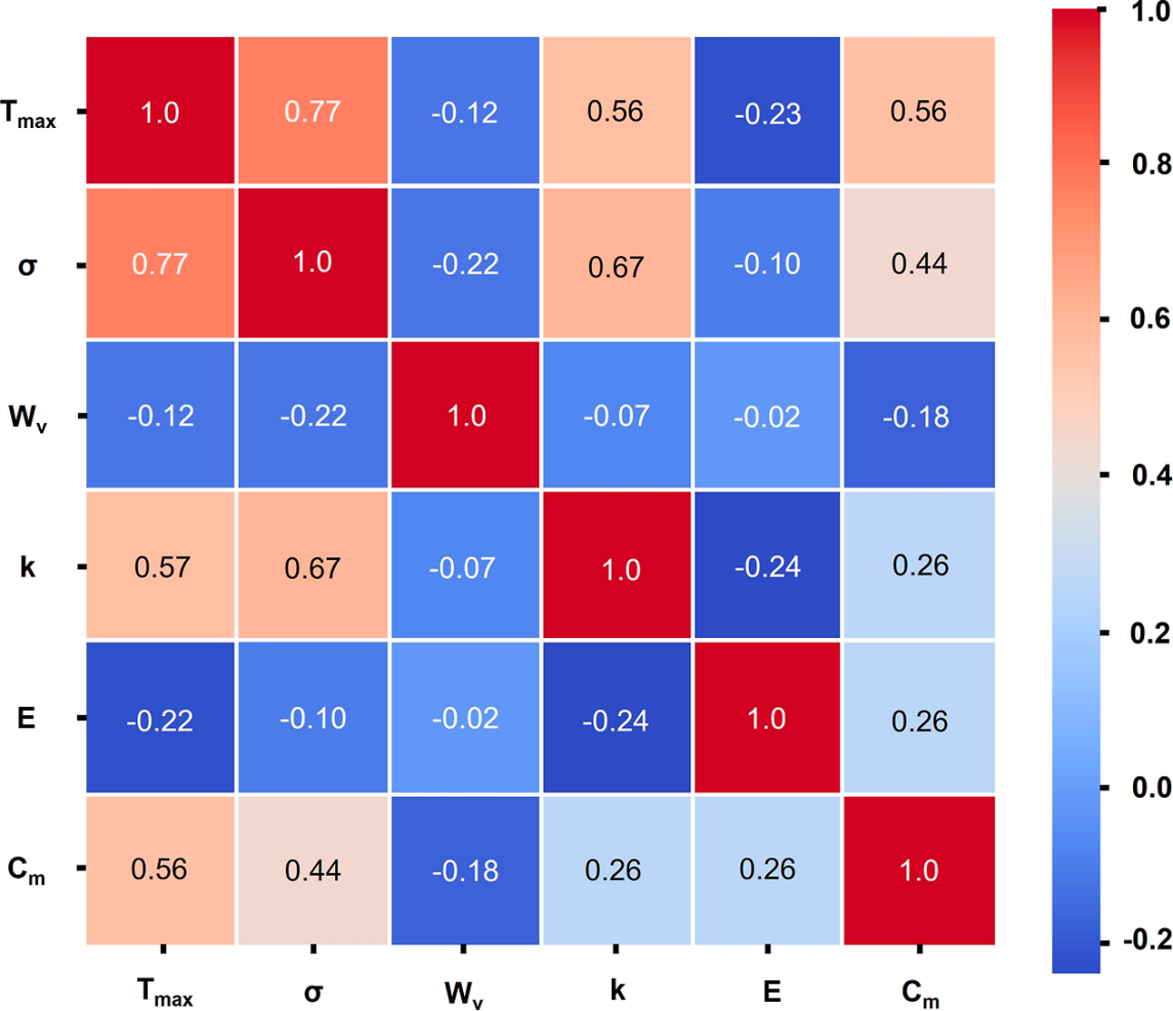
Pearson correlation matrix “*r*” for six material properties across 22 MD candidate materials. Cells display “*r*”; the color scale spans ([-1, +1]). *P*-values were computed for all 15 pairwise comparisons and evaluated with Bonferroni correction. Significant correlations: *T*_max_ and σ have *r* = 0.67. Not significant after correction but notable: *T*_max_ and *C*_m_ have *r* = 0.56. *W*_v_ and *E* show weak associations with other properties of |*r*|<0.3, indicating limited dependence on bulk material chemistry.

#### 3.4.3 Performance Index Framework

To enable systematic multicriteria material ranking, a normalized performance index (Π) was formulated incorporating thermal stability, mechanical strength, vapor transport capability, thermal conductivity, and environmental sustainability with their normalized values:

4 $$\Pi =\omega_{T_{\max}}\left(T_{norm}\right)+\omega_{\sigma }\left(\sigma_{norm}\right)+\omega_{W_{v}}\left(W_{v_{norm}}\right)+\omega_{k}\left(k_{norm}\right)+\omega_{S}\left(S_{norm}\right)$$

where *T*_max_ is the maximum temperature (°C), σ is the tensile strength (MPa), W_v_ is the water vapor transmission rate (g·mm·m^–2^·day^–1^), k is the thermal conductivity (W·m^–1^·°C^1–^), and S is a sustainability factor, quantified by combining the effects of embodied energy and CO_2_ emissions:

5 $$S_{norm}=\frac{1}{2}\left(E_{norm}+C{O_{2}}_{norm}\right)$$

ω is the corresponding weight for each factor. Here, the overall sustainability index (*S*_norm_) was calculated as the arithmetic mean of normalized embodied energy and CO_2_ emission values. The analysis of Π for different membrane materials is done based on three criteria: (i) equal importance, (ii) high flux priority, and (iii) sustainability priority. Thermal conductivity is reverse-normalized, as lower *k* is preferred, as shown in eq S3. The normalization of parameters along with weights for specific criteria is discussed in Section S5 of Supporting Information.

Under equal-importance weighting, a balanced significance was assigned to thermal, mechanical, transport, and sustainability attributes. The calculated Π data are reported in Figure 10a. Candidates that showcased the highest overall Π are titania (0.67), zirconia (0.63), SBS (0.55), and alumina (0.46), reflecting the dominating effects of parameters like excellent thermal stability and vapor-transport capability for candidates. Ceramics such as alumina, titania, and zirconia achieved a higher ranking due to their significant thermal tolerance and high mechanical strength, which strongly influence the composite index despite their relatively lower water vapor permeability. Remarkably, the elastomeric polymer achieved the highest Π in this category primarily due to its exceptional water vapor flux of ∼268 g·mm·m^–2^·day^–1^, which offsets its lower mechanical robustness. On the other hand, conventional polymers such as PE, nylon, PVDF, and ABS got a lower value of index, Π = 0.29–0.40, reflecting their moderate flux and lower strength. Overall, this approach emphasizes that the candidates combining mechanical and thermal resilience with reasonable vapor transport are preferred, while ceramics maintain dominance via intrinsic thermal and mechanical superiority even under lower sustainability. Weighting scenarios reflect typical industrial priorities: flux-dominant applications (ω_Wv_ = 0.6) represent desalination where productivity drives economics; sustainability-dominant (ω_S_ = 0.6) reflects regulatory-constrained contexts. Future work should engage industry stakeholders to refine application-specific weightings through multicriteria decision analysis.

**Figure 10:**
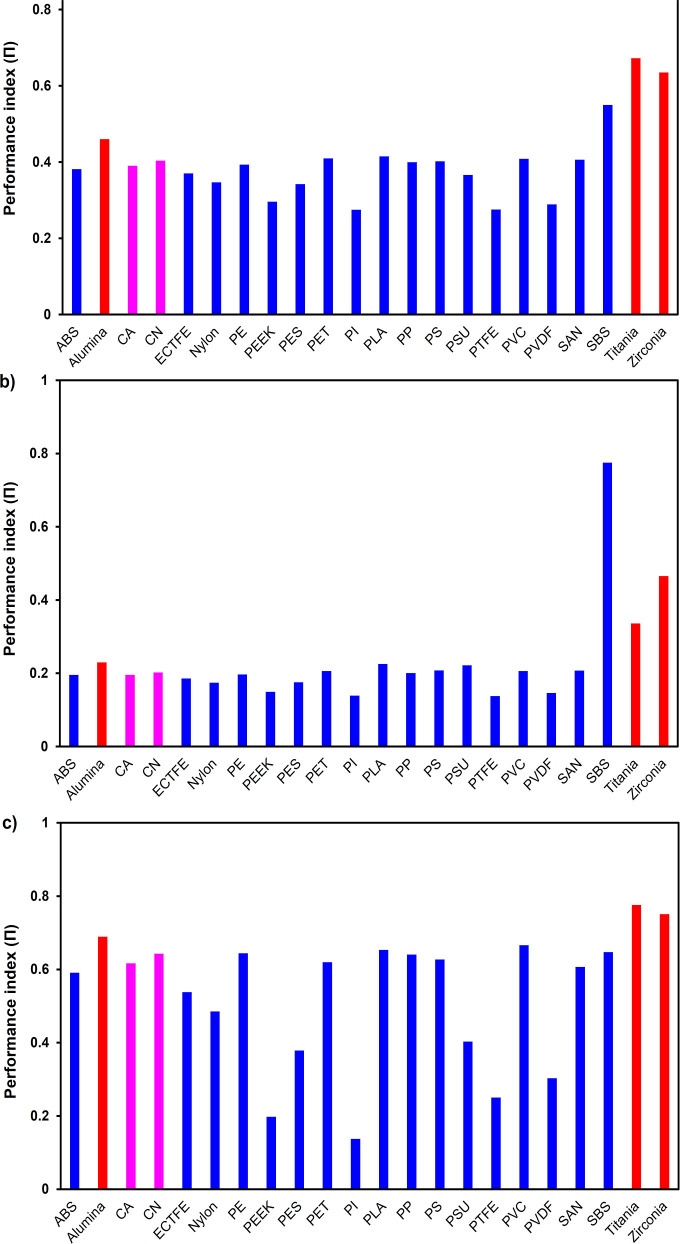
Normalized multicriteria (Π) for candidate membrane materials under three weighting schemes. (a) Equal importance: top performers are titania with Π = 0.67, zirconia with 0.63, SBS with 0.55, and alumina with 0.46, reflecting the strong influence of thermal stability and strength; (b) flux priority: SBS leads decisively with Π = 0.77, while ceramics drop Π < 0.50 due to intrinsically lower permeability; (c) sustainability priority: low-footprint commodity polymers PVC got Π = 0.67, PE (0.64), and PP (0.64) performed the best. Collectively, the rankings expose the trade-offs between transport, thermo-mechanical robustness, and environmental impact, with the preferred materials shifting according to application priorities. With a focus on vapor flux, materials having high permeability rapidly improved the overall Π as shown in (b): SBS improved its performance (Π = 0.77) due to excellent water-vapor flux, outperforming all other materials. Interestingly, ceramics exhibited a decline in ranking (Π < 0.50) owing to their intrinsically lower vapor flux. These results highlight the importance of flux strongly benefiting polymeric materials with high permeability, reinforcing the trade-off between the mechanical robustness and transport ability inherent in membrane materials. All other candidates have a significant drop in the index (Π < 0.25) due to the lower value of water-vapor flux. Apart from SBS, other polymers with higher permeability like PLA (Wv = 9.67 g·mm·m-2·day-1) and PSU (*W*_v_ = 20.98 g·mm·m-2·day-1) were ranked fifth and sixth, respectively. The improvement in Π for such polymers emphasizes how improved vapor flux properties can outweigh the moderate or low thermal and mechanical characteristics under this weighting approach.

The dominance of SBS under flux-priority weighting merits critical evaluation, as its anomalously high *W*_v_ value of 310 g·mm·m^–2^·day^–1^ exerted a disproportionate influence on the normalized Π calculation. When SBS is excluded from the normalization pool as a sensitivity check (equal-weighting scenario), PSU emerges as the top-ranked polymer (Π = 0.55), followed by PLA (Π ≈ 0.41), with commonly deployed materials PE, PVDF, and PP achieving competitive Π values in the 0.20–0.30 range. The relatively lower Π for PE and PVDF under flux-priority weighting reflects their moderate intrinsic *W*_v_ values rather than operational inadequacy. These materials compensate through excellent chemical compatibility across diverse feed environments demonstrated in Figures 4 and 5, proven fabrication track records enabling consistent membrane production, and favorable sustainability profiles shown in Figure 10c. This further corroborates that material rankings are strongly scenario-dependent, and commonly deployed polymers remain highly competitive when multidimensional deployment considerations are incorporated. It is acknowledged that SBS’s exceptionally high intrinsic water vapor transmission rate, *W*_v_∼ 310 g·mm·m^–2^·day^–1^, functions as the normalization maximum for the *W*_v_ parameter, which compresses relative flux scores for all remaining materials under min–max scaling. To assess the robustness of Π rankings to this outlier effect, a sensitivity analysis was conducted excluding SBS from the normalization pool (*n* = 21); full results are reported in Table S5 of the Supporting Information. The analysis confirms that ceramic rankings (titania Π = 0.67, zirconia Π = 0.63) and sustainability-priority rankings are insensitive to SBS exclusion with rank changes of 0–2 positions for most materials. The most significant redistribution occurs for PSU, which advances 11 rank positions (Π = 0.55, equal weighting) when SBS is excluded. Under flux-priority weighting with SBS excluded, PSU achieves Π = 0.775, becoming the top-ranked polymer and the most practically deployable high-flux alternative given SBS’s mechanical limitations. These results corroborate that the framework’s principal material recommendations are structurally robust rather than artifacts of a single outlier property value.

When sustainability is given the highest importance, combining the embodied energy and CO_2_ emissions, materials with lower environmental impact observed significant improvement in their Π as shown in Figure 10c. Commodity polymers like PVC (Π = 0.67), PE (Π = 0.64), and PP (Π = 0.64) improved their ranking due to their low carbon footprint and being less energy intensive, while titania, PET, and SAN went out of the top 10 candidates when sustainability was given priority owing to increased energy cost and CO_2_ emissions, along with the other parameters being moderate in improving the index. This trend reflects the importance of integrating the environmental factors into material selection, as the inclusion of the sustainability factors leads to ranking from just high-performance toward eco-efficient polymer alternatives. The multicriteria Π framework functions as a decision support tool that makes material trade-offs explicit and quantifiable to enable practitioners to match material selection to their specific feed chemistry, throughput requirements, and sustainability constraints using the application guidance consolidated in the Quick Selection Guide above. The three weighting scenarios evaluated, equal importance (Π = 0.67 for titania), flux priority (Π = 0.77 for SBS), and sustainability priority (Π = 0.77 for PVC), demonstrate that optimal material selection is context-dependent and shifts substantially based on operational priorities and application constraints. For desalination in water-scarce regions with inexpensive thermal energy, flux-maximizing polymers such as SBS may dominate; conversely, resource recovery from high-value waste streams in stringent regulatory environments may prioritize chemically robust ceramics or fluoropolymers despite lower flux. The framework’s principal value lies in making these trade-offs explicit and quantifiable, enabling stakeholders to transparently evaluate material options against their specific technical, economic, and sustainability requirements rather than applying a single prescriptive recommendation across all circular water economy contexts. This context-sensitivity has a direct policy implication: procurement specifications for industrial MD systems should define operational priority weights alongside material requirements, rather than specifying a single material class, to ensure deployment decisions are aligned with facility-specific sustainability and performance objectives. Table 2 consolidates the multicriteria Π analysis into an application-specific decision matrix, identifying the top three membrane materials for each of eight distinct deployment contexts alongside critical material exclusions and primary selection drivers.

**Table 2:** Application-Specific Material Selection Guide for Membrane Distillation: Top Three Candidates across Eight Deployment Contexts

application context	rank 1	π/key property	rank 2	π/key property	rank 3	π/key property	critical exclusion	selection driver
standard desalination	PTFE	Π = 0.4; exp validated 29 ± 4 kg·m^–2^·h^–1^; intrinsic hydrophobic	PP	Π = 0.37; exp validated 14 ± 2 kg·m^–2^·h^–1^; lowest cost	PVDF	Π = 0.35; exp validated 11 ± 3 kg·m^–2^·h^–1^; robust aqueous	SAN, PLA, PSU (saltwater: acceptable only; flux decline risk)	flux + saltwater compatibility
organic solvent wastewater	PTFE	Π = 0.4; excellent organic resistance; Exp validated flux	ECTFE	Π = 0.41; excellent organic + universal pH; fluoropolymer	PP	Π = 0.37; excellent organic; low cost; intrinsic hydrophobic	SAN, PS, ABS, SBS, CA, PSU, PES (organic: unacceptable)	organic solvent resistance
alkaline treatment (pH > 12)	PTFE	Π = 0.4; excellent alkali and flux; > 99% salt rejection	PEEK	Π = 0.51; excellent alkali; *T*_max_ = 250 °C; high strength	PE	Π = 0.40; excellent alkali; low cost; acid acceptable	PLA, CA, PI (alkaline: unacceptable; rapid hydrolysis)	alkali resistance + flux
acid treatment (pH < 3)	PTFE	Π = 0.4; excellent acid and alkali; C–F bond strength	PVDF	Π = 0.40; excellent acid; Pharma track record; good flux	ECTFE	Π = 0.41; excellent acid and organic; fluoropolymer	PET, SBS, CA, PLA, nylon, PEEK (acid: unacceptable)	acid resistance (C–F bond strength)
food-grade processing	PTFE	Π = 0.44; FDA/USP Class VI; Exp validated; zero extractables	PP	Π = 0.37; food-grade grades; Exp validated; cost-effective	PE	Π = 0.40; FDA polyolefin; low extractables; low cost	PVC, ABS, SBS (not food-approved; extractables risk)	regulatory compliance
pharmaceutical/medical grade	PTFE	Π = 0.44^1^; USP Class VI; ISO 10993; widest pharma use	PVDF	Π = 0.40; Pharma-grade grades; good flux; acid stable	PEEK	Π = 0.51; biocompatible; *T*_max_ = 250 °C; chemically robust	PVC, ABS, SBS, PLA (no USP/ISO 10993 certification)	biocompatibility (ISO/USP)
high-temperature industrial (>200 °C)	titania	Π = 0.67; *T*_max_ > 1000 °C; best overall balanced Π	zirconia	Π = 0.63; *T*_max_ > 1000 °C; acid acceptable	alumina	Π = 0.46; *T*_max_ > 1000 °C; highest strength (200–400 MPa)	PS, ABS, SBS, PVC, PLA (<100 °C *T*_max_; catastrophic failure)	thermal stability (*T*_max_)
sustainability-constrained	PVC	Π = 0.67; lowest CO_2_ and energy; alkali + acid √	PE	Π = 0.64; low embodied energy; excellent alkali; acid acceptable	PP	Π = 0.64; low embodied energy; excellent alkali; exp validated	PI, PEEK, PTFE, PES (highest embodied energy and CO_2_)	LCA sustainability (Π, CO_2_, energy)

Across all eight contexts, PTFE and alumina appear among the top three in five or more categories, confirming their near-universal suitability; however, sustainability-constrained deployments should prioritize PVC, PE, or PP, which achieve Π = 0.64–0.67 under sustainability-priority weighting while maintaining adequate flux and chemical resistance for aqueous applications.

### 3.5 Surface Energy and Fabrication Readiness

Hydrophobicity is a non-negotiable functional requirement for MD membranes: the vapor–liquid interface at the pore mouth must resist feed-side wetting to maintain separation integrity, and contact angle directly governs the liquid entry pressure (LEP) threshold below which feed breakthrough occurs. Across the 22 candidate materials, surface energy characteristics stratify into three fabrication-readiness tiers that have direct consequences for deployment cost and process complexity. The majority of conventional thermoplastic polymers including ABS, ECTFE, PEEK, PET, PE, PLA, PP, PVDF, PTFE, PS, PSU, PVC, SAN, and SBS possess intrinsically low surface energy characteristics favorable for MD, requiring minimal to negligible postfabrication surface modification to achieve the contact angles (>90°) and LEP values (>250 kPa) needed for reliable vapor–liquid separation. This intrinsic hydrophobicity substantially reduces fabrication complexity and processing cost relative to materials requiring post-treatment and is reflected in the Table 1 contact angle values of 105°, 96°, and 123° for the commercially tested PP, PVDF, and PTFE membranes, respectively, all achieved without surface modification. PES, nylon, and PI represent notable exceptions within the engineering polymer class: despite conventional polymeric chemistry, these materials exhibit suboptimal hydrophobicity and require surface treatment before MD deployment. Applicable strategies include hydrophobic coating deposition (fluorosilane, PDMS, or PTFE dispersion), chemical grafting of low-surface-energy moieties via plasma activation followed by silane coupling, or incorporation of hydrophobic nanoparticle additives during phase inversion. Bioplastic and ceramic materials universally require surface modification to achieve requisite hydrophobicity, introducing additional processing complexity and cost that should be factored into any total cost of ownership comparison against naturally hydrophobic polymers.

Beyond intrinsic surface energy, practical MD performance depends on the system architecture that the material selection framework does not encode. Module geometry, flat sheet versus hollow fiber versus spiral wound, determines packing density, flow channel hydrodynamics, and boundary layer thickness, each of which modulates the effective transmembrane temperature gradient and therefore vapor pressure driving force independently of membrane material. Feed and permeate temperatures, flow velocities, feed concentrations, and thermal recovery configuration collectively set the operating envelope within which material properties are expressed. The implication for framework users is that the Π rankings identify the material that maximizes multicriteria potential under standardized conditions; realizing that potential in a specific facility requires subsequent integration with fabrication process optimization and module engineering. The strong *R* ^2^ ≈ 1 agreement between Ansys Granta predictions and experimental flux validates the informatics framework for material screening under controlled conditions, but experimental validation at the module scale remains essential as surface modification, membrane morphology, and module hydrodynamics are finalized for each candidate material. Future iterations of the Π framework should incorporate surface modification complexity as a sixth criterion, penalizing materials requiring multistep postfabrication treatment to make fabrication readiness an explicit rather than implicit component of the selection index.

## 4 Discussion

The application of Ansys Granta EduPack to thermally driven membrane separation appears to be without precedent in the published literature. Prior applications of Granta informatics have been confined predominantly to structural materials selection, lightweight design, and packaging sustainability assessment. The transfer of this platform to MD—a thermally, mechanically, and chemically demanding separation context—demonstrates that commercially available materials databases can serve as a primary discovery tool for functional membrane materials without requiring purpose-built computational infrastructure, lowering the barrier for systematic material screening across research groups globally.

### 4.1 Benchmarking against Published MD Studies

The experimental fluxes obtained here for PP at 14 ± 2, PVDF at 11 ± 3, and PTFE at 29 ± 4 kg·m^–2^·h^–1^ at a 70/20 °C feed/permeate temperature differential with 35 g·L^–1^ NaCl were consistent with the range reported in the broader DCMD literature for membranes of comparable pore size and porosity. Published PTFE flux values at similar operating conditions typically span 10–45 kg·m^–2^·h^–1^ depending on membrane thickness, porosity, and module design, placing the 29 ± 4 kg·m^–2^·h^–1^ obtained here squarely within the mid-to-upper range expected for a 0.45 μm, 77% porosity membrane. The PP flux of 14 ± 2 kg·m^–2^·h^–1^ likewise aligns with literature reports for commodity PP membranes at 60–65% porosity, which consistently underperform PTFE due to lower intrinsic hydrophobicity and slit-like pore geometry reducing effective vapor transport area. The PVDF result of 11 ± 3 kg·m^–2^·h^–1^ is somewhat lower than the upper range reported for PVDF in the literature (15–25 kg·m^–2^·h^–1^), likely attributable to the phase-inversion sponge-like pore network of the Tisch Scientific membrane imposing higher tortuosity than electrospun or stretched PVDF alternatives. Critically, the rank order PTFE > PP > PVDF reproduced by Ansys Granta-derived predictions (*R* ^2^ ≈ 1) is consistent with the relative ordering reported by independent studies using different module geometries and feed compositions, supporting the generalizability of the informatics screening approach beyond the specific experimental conditions of this study. The performance index Π values for titania (0.67 under balanced weighting), SBS (0.77 under flux priority), and PVC (0.77 under sustainability priority) represent the first multicriteria composite rankings across a 22-material portfolio spanning ceramics, engineering polymers, and biopolymers simultaneously; prior studies optimizing for single criteria would not have identified titania as a balanced-weighting leader, nor PVC as a sustainability-priority candidate, demonstrating the nonobviousness and added value of the multicriteria formulation.

### 4.2 Framework Limitations

Three limitations warrant explicit acknowledgment to correctly bound the scope of the framework’s conclusions. First, Ansys Granta bulk property data reflect homogeneous, fully dense material specimens; fabricated MD membranes deviate from bulk in porosity, effective thickness, crystallinity, and surface energy as a direct consequence of phase inversion, biaxial stretching, electrospinning, or sintering. The *R* ^2^ ≈ 1 agreement observed here validates relative ranking under controlled laboratory conditions using membranes of identical pore size and thickness, but absolute flux prediction for membranes with different morphological parameters would require fabrication-specific corrections to eq 2. Second, the LCA scaling assumes a conservative seven-day membrane operational lifespan; this assumption drives the high annual replacement mass (1150 lb) that amplifies production-phase environmental burdens. Real industrial MD systems routinely achieve membrane lifespans of 30–180 days before replacement, and sensitivity analysis across this range would progressively favor high-embodied-energy materials with strong recycling pathways, a refinement needed before the sustainability rankings can be used directly in facility procurement decisions. Third, experimental validation was conducted exclusively with a 35 g·L^–1^ NaCl feed under steady-state conditions; real industrial waste streams introduce fouling precursors, scaling ions, surfactants, and pH variability that alter effective flux, salt rejection, and long-term membrane wettability in ways that the current informatics framework does not capture. Chemical compatibility classifications from Ansys Granta represent equilibrium immersion-test ratings rather than dynamic fouling or long-term degradation assessments and should be interpreted as candidate elimination tools rather than predictors of operational durability. Future iterations should integrate minimum threshold constraints, for instance, automatic disqualification of materials with tensile strength below 10 MPa or with less than “acceptable” ratings in the target feed environment to prevent mechanically or chemically marginal candidates such as SBS from appearing competitive in composite Π rankings.

### 4.3 Circular Water Economy Policy Implications

The framework generates three actionable implications for policy and industrial deployment within a circular water economy. First, the saltwater–organic solvent compatibility asymmetry identified in Section 3.2 establishes that feed chemistry, not flux ranking or material cost, should serve as the primary filter in any material selection decision for industrial MD systems. Environmental permitting of MD systems treating mixed organic-aqueous effluents such as pharmaceutical mother liquors, semiconductor rinse waters, and mining leachates increasingly requires documentation of membrane chemical compatibility across the full feed composition range; the Ansys Granta multienvironment compatibility profile demonstrated here provides precisely this evidence at the prefabrication screening stage, enabling regulatorily defensible material choices before capital is committed. Second, the finding that production-phase environmental impact alone is an insufficient selection criterion illustrated by PEEK and PES achieving net lifecycle benefits through recycling pathways despite high embodied energy has direct relevance to emerging extended producer responsibility legislation and ISO 14040-compliant environmental product declarations. Procurement specifications for industrial MD systems should require full cradle-to-gate plus end-of-life lifecycle documentation, not merely embodied carbon, to avoid systematically disadvantaging high-performance recyclable materials relative to low-cost, nonrecyclable alternatives. Third, the weighting-scenario structure of the Π framework maps directly onto the regulatory and operational priorities that differ across deployment contexts: water-scarce regions with access to low-grade waste heat and high desalination throughput requirements are best served by flux-maximizing candidates, while facilities operating under stringent environmental discharge regulations or recovering high-value minerals from aggressive waste streams should weight sustainability and chemical universality. Codifying these context-specific weighting schemes into publicly available decision tools analogous to existing membrane selection databases for reverse osmosis would lower the barrier for adoption of systematic material selection across the diverse landscape of circular water economy implementations.

## 5 Conclusion

This study establishes the first integrated, replicable framework combining materials informatics, experimental validation, and LCA for evidence-based membrane material selection in MD, directly addressing the absence of systematic multidimensional selection methodologies identified in the literature. The framework demonstrates unambiguously that optimal material selection cannot be reduced to a single-criterion optimization; instead, it depends on a complex interplay among technical performance, chemical compatibility, environmental burden, and end-of-life recovery potential that shifts meaningfully with operational context. Three principal technical insights emerge. First, Ansys Granta-derived bulk property data correctly rank commercial membranes by relative performance (*R* ^2^ ≈ 1), confirming that database-driven screening constitutes a reliable, resource-efficient first stage of materials discovery for thermally driven separations provided rankings are interpreted comparatively rather than as absolute flux predictors. Second, the cross-property correlation structure reveals that thermomechanical robustness and vapor transport operate as near-independent axes, meaning no single material class dominates across all performance dimensions simultaneously; this orthogonality is precisely what necessitates the multicriteria framework rather than heuristic selection. Third, lifecycle sustainability analysis inverts the intuitive hierarchy: materials with the highest production-phase environmental burden (PEEK, PES, and PI) achieve net lifecycle benefits through established recycling infrastructure, challenging the convention that production carbon footprint alone determines material environmental performance. The chemical compatibility analysis reveals an equally important asymmetry: alkaline and acidic resistance arise from distinct degradation mechanisms, rendering generalized chemical resistance classifications insufficient for deployment decisions. Only fluoropolymers (PVDF, PTFE, and ECTFE) and alumina maintain stability across both pH extremes, positioning them as the only truly universal candidates for industrial contexts with variable feed chemistry. This finding carries direct policy relevance: environmental regulations governing industrial wastewater discharge increasingly demand treatment of streams with dynamic pH profiles, and material selection decisions made at the design stage determine whether MD systems can operate reliably across these conditions without premature membrane replacement.

The multicriteria Π operationalizes these trade-offs into a transparent decision-support tool. Material rankings shift substantially across the three weighting scenarios: titania under balanced criteria (Π = 0.67), SBS under flux priority (Π = 0.77), and PVC under sustainability priority (Π = 0.77), reflecting the context-dependence inherent to industrial deployment rather than any ambiguity in the framework itself. This flexibility positions the framework as a platform adaptable to the full heterogeneity of circular water economy applications: from commodity desalination, where flux economics dominate, to regulated resource recovery contexts, where lifecycle sustainability and chemical durability carry greater weight.

In aggregate, this work delivers three actionable contributions to the environmental science and engineering community: (1) validation that materials informatics databases can replace costly trial-and-error experimentation for initial membrane material screening, reducing development timelines while maintaining rigorous experimental grounding through strategic validation campaigns; (2) a transparent multicriteria framework that captures the complex interplay among technical performance, chemical compatibility, and lifecycle sustainability routinely obscured by single-parameter optimization; and (3) application-specific material recommendations spanning conventional desalination through specialized resource recovery from chemically aggressive industrial waste streams including the identification of eight universal materials suitable for saltwater, organic solvent, and extreme pH environments, 12 candidates for aqueous-only applications, and three ceramic alternatives for extreme-temperature contexts exceeding 400 °C. Together, these contributions position the framework as a practical decision-support tool for accelerating circular water economy implementation across diverse industrial sectors. The methodology transfers directly to other thermally driven separations (pervaporation, osmotic distillation, and thermopervaporation) and provides a scalable foundation for progressive enhancement. Near-term priorities include validation against real industrial waste streams of variable chemistry, sensitivity analysis of Π rankings across extended membrane lifespans (30–180 days), and machine learning integration to correlate structural features with separation performance. Incorporating technoeconomic metrics into the performance index and conducting long-term field trials will further strengthen the evidence base for deploying MD as a mainstream circular water economy technology, one capable of simultaneously recovering critical minerals, nutrients, and purified water from diverse waste streams while reducing freshwater extraction pressure and supporting industrial supply chain resilience.

To the authors’ knowledge, this is the first quantitative lifecycle inversion demonstration for membrane materials evaluated under matched industrial-scale facility conditions using a standardized life cycle inventory (LCI) database, establishing a methodological precedent for lifecycle-complete material selection in thermally driven separations. The Ansys Granta platform, applied here for the first time to thermally driven membrane separation, provides a commercially accessible, continually updated materials intelligence infrastructure that research groups and industrial practitioners can adopt without purpose-built computational resources. This positions materials informatics as a practical first-stage discovery tool for the broader membrane engineering community, not only for MD but also for pervaporation, osmotic distillation, and emerging hybrid separation processes.

## Supplementary Material



## Data Availability

All data supporting this study are available on https://github.com/anjurgupta/Membrane-Materials-Optimization-MD.

## Notes

Materials selection framework and systematic screening protocol; regulatory compliance assessment: food contact and medical grade certification; end-of-life energy and CO2 offset potential; custom-designed membrane distillation (MD) setup; calculations of material performance analysis; and economic feasibility and cost–performance trade-offs (PDF)
