# Supplementary material for: Systematic Material Optimization for Membrane Distillation Resource Recovery through Materials Informatics, Life Cycle Assessment, and Industrial Scalability
Source: ACS ES T Eng. 2026 Apr 10;6(5):1571–92. doi: 10.1021/acsestengg.6c00062 (PMC13162256; doi:10.1021/acsestengg.6c00062)
Supplement: Supplementary file 1 [file ee6c00062_si_001.pdf]

Supporting Information

Systematic Material Optimization for Membrane Distillation Resource Recovery Through Materials Informatics, Life Cycle Assessment, and Industrial Scalability

Saketh Merugu[†], Keval Bharatbhai Suthar[†], and Anju Gupta[†] [*]

[†]Department of Mechanical, Industrial and Manufacturing Engineering, The University of Toledo, 2801 West Bancroft Street, Toledo, OH 43606

*Corresponding author:

Dr. Anju Gupta

E-mail: anju.gupta@utoledo.edu

Department of Mechanical, Industrial and Manufacturing Engineering, University of Toledo, 1610 N Westwood Ave, NI 4055, Toledo, OH 43606.

Telephone: +1 (413) 530-8213

S1. Materials Selection Framework and Systematic Screening Protocol

A hierarchical multi-stage filtering protocol was employed to systematically identify candidate membrane materials from the comprehensive Ansys Granta database. The initial search query, executed using Granta's Advanced Search function, identified all materials matching criteria: 1) material type classification as thermoplastic polymers, biopolymers, or inorganic ceramics; 2) processing compatibility with membrane fabrication methods including melt-processable, solution-castable, or slurry-formable; 3) sufficient data availability for key material properties such as thermal stability, tensile strength, density. This initial automated search yielded 216 candidate materials distributed across Polymers including 168 materials, 22 biopolymers, and 26 ceramics. From this initial pool of 216 candidates, systematic multi-criterion filtering was applied sequentially using Granta's Multi-Criteria Filter function to progressively narrow the candidate pool according to membrane-specific performance requirements. Stage 1: Thermal stability filter retained materials with maximum service temperature ≥50°C, eliminating 6 materials that were primarily low-temperature elastomers incompatible with MD operation. Stage 2: Mechanical integrity filter retained materials with tensile strength ≥5 MPa, eliminating 21 soft elastomers materials. Stage 3: Commercial availability and economic viability retained materials with production volume >1000 metric tons/year and unit cost <$50/kg, eliminating 17 specialty research materials and custom-synthesized polymers. Stage 4: Application history filter retained materials with documented prior use in filtration, separation, or water treatment applications per Granta's application database and data completeness filter retained materials with ≥80% availability of critical properties required for comprehensive multi-criteria analysis including thermal stability, mechanical strength, density, cost, embodied energy, water vapor transmission, chemical resistance, eliminating 100 materials without relevant separation technology history and

50 materials with data gaps. This sequential filtering resulted in a final cohort of 22 materials representing a balanced portfolio spanning all material classes: commodity polymers (PE, PVC, PP, PS, PET, ABS, SBS, SAN, PLA, representing 9 materials), advanced polymers (Nylon, PVDF, PTFE, PSU, PES, PEEK, ECTFE, PI representing 8 materials), biopolymers (CN, CA, representing 2 materials), and ceramics (alumina, zirconia, titania, representing 3 materials). This stratified final selection ensured representation across the complete performance spectrum of thermal stability: 50–2400°C; tensile strength: 7–250 MPa; embodied energy: 0.3–9.0 $\times 10^6$ MJ/year while maintaining economic feasibility and commercial availability essential for industrial-scale MD deployment.

Table S1. List of candidate materials selected for this study along with its categories.

Category	Candidate Membrane Material
Polymers	Polyethylene (PE)[24]
	Polyethylene Terephthalate (PET)[25]
	Polyvinylidene Fluoride (PVDF)[26]
	Polytetrafluoroethylene (PTFE)[27]
	Polyvinyl Chloride (PVC)[28]
	Polypropylene (PP)[28]
	Polystyrene (PS)[29]
	Acrylonitrile Butadiene Styrene (ABS)[30]
	Styrene-Acrylonitrile Copolymer (SAN)[31]
	Styrene Butadiene Styrene (SBS)[32]
	Polysulfone (PSU)[33]
	Polyether Sulfone (PES)[33]
	Ethylene Chlorotrifluoroethylene Polyetherimide (ECTFE)[34]
	Polyetheretherketone (PEEK)[35]

	Polylactic Acid (PLA)[36]
	Polyamide (PA12, Nylon)[26]
	Polyimide (PI)[36]
Biopolymers	Cellulose (CN)[37]
	Cellulose Acetate (CA)[38]
Ceramics	Alumina[39]
	Titania[40]
	Zirconia[41]

S2. Regulatory Compliance Assessment: Food Contact and Medical Grade Certification

Material eligibility for specialized applications involving direct contact with consumables or biological systems requires compliance with stringent regulatory frameworks governing chemical safety, extractables/ leachables profiles, and biocompatibility. Figure S1 presents systematic classification of membrane materials according to food contact approval and medical grade certification status, stratifying candidates based on regulatory compliance data extracted from the Ansys Granta materials database and cross-referenced with FDA, EU, and ISO standards. Membrane materials employed in food and beverage processing, pharmaceutical purification, potable water production, and nutraceutical manufacturing must meet rigorous food contact safety standards to prevent migration of potentially harmful chemical species into products intended for human consumption. Regulatory frameworks governing food contact materials include FDA Title 21 CFR (United States), EU Regulation 10/2011 and Framework Regulation 1935/2004 (European Union), and national food safety standards in other jurisdictions. These regulations establish maximum permitted migration limits for specific substances, require toxicological safety assessments, and mandate compositional restrictions on additives, plasticizers, and residual monomers. As demonstrated in Figure S1, the majority of evaluated membrane materials have

achieved food contact approval, categorized as "Yes" (unconditional approval for food contact applications) or "Conditional" (approved with restrictions on temperature, contact duration, or food type). Materials designated as "Yes" for food contact include commodity polymers (polyvinyl chloride (PVC), polyethylene (PE), polyethylene terephthalate (PET), styrene-acrylonitrile copolymer (SAN), styrene-butadiene-styrene (SBS), polypropylene (PP), polystyrene (PS), acrylonitrile butadiene styrene (ABS), polylactic acid (PLA)), bioplastics (polyamide materials (PA12/Nylon)), high-performance polymers (polysulfone (PSU), polyvinylidene fluoride (PVDF), polytetrafluoroethylene (PTFE), poly(ethyl chlorotrifluoroethylene) (ECTFE), polyetheretherketone (PEEK)), and ceramic materials (zirconia, titania, alumina). These materials have undergone extensive toxicological evaluation and migration testing, demonstrating compliance with regulatory migration limits and absence of substances of concern in food contact scenarios. The "Conditional" designation was not assigned to any materials in the current evaluation, though it should be noted that certain formulations or grades of approved polymers may carry usage restrictions. For example, some PVC formulations are restricted from high-temperature or fatty food contact due to plasticizer migration concerns, and certain grades of polyamides may be limited to specific temperature ranges or contact durations. The absence of materials in the conditional category in this assessment reflects the use of standard food-grade polymer formulations represented in the Ansys Granta database. Only two materials received "No" classification for food contact applications: cellulose (CN) and cellulose acetate (CA). While cellulose-based materials are naturally derived and generally recognized as safe for incidental contact, the specific CN formulations evaluated lack formal food contact approval due to potential presence of processing additives, residual chemicals from pulp treatment, or insufficient regulatory submission data. Cellulose acetate similarly lacks universal food contact certification, though

specific pharmaceutical-grade or food-grade CA formulations may exist with appropriate regulatory approval. These materials are precluded from deployment in any membrane distillation application where the permeate is intended for direct human consumption or food processing use without additional regulatory qualification and testing. The broad food contact approval observed across most polymer and ceramic classes reflects several decades of regulatory evaluation and commercial deployment in food packaging, beverage containers, food processing equipment, and potable water systems.

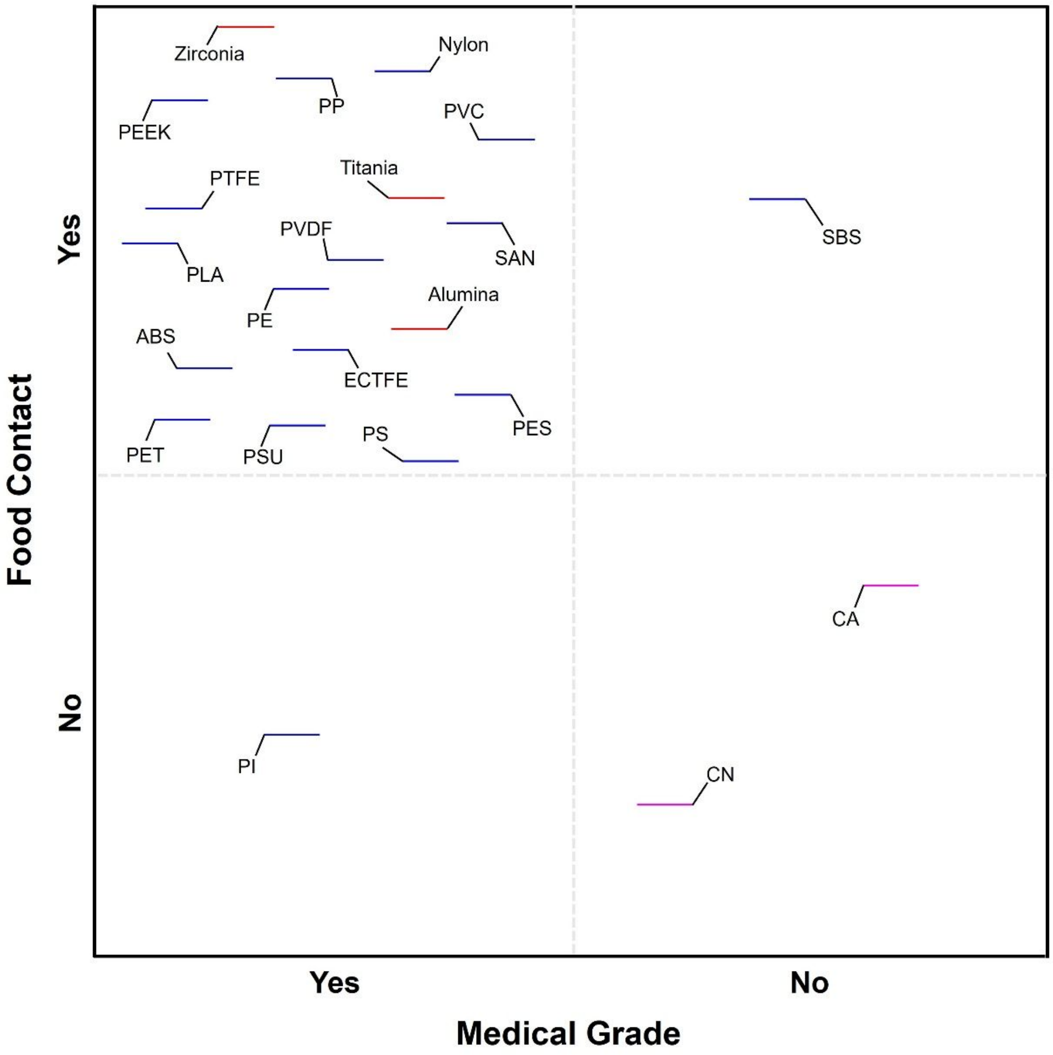

Figure S1. Two-tier compliance assessment according to (a) food contact approval status (Yes/Conditional/No) and (b) medical grade certification (True/False). Nearly all polymers and ceramics achieve food contact approval; cellulose-based materials (CN, CA) lack food contact certification. Medical grade certification achieved by commodity polymers (PVC, PE, PET, PP), high-performance fluoropolymers (PVDF, PTFE, PEEK), and ceramics (zirconia, alumina); styrene-based polymers (PS, SBS) and excluded cellulosics. Classifications based on regulatory frameworks including FDA 21 CFR, EU Regulations 10/2011 and 2017/745, and ISO 10993 biocompatibility standards. Compliance data extracted from Ansys Granta materials database.

Medical grade material certification imposes even more stringent requirements than food contact approval, encompassing biocompatibility testing (ISO 10993 series), chemical characterization, extractables/leachables profiling, sterility compatibility, and compliance with medical device regulations (FDA 21 CFR Part 820, EU Medical Device Regulation 2017/745). Medical grade materials must demonstrate absence of cytotoxic, genotoxic, or sensitizing responses in biological systems and maintain chemical stability through sterilization processes including autoclave, gamma irradiation, ethylene oxide. Fig S1 reveals that a substantial subset of materials achieve medical grade certification, classified as "True" for medical grade eligibility. Materials meeting medical grade standards include commodity polymers PVC, (PE), PET, SAN, PP, ABS, PLA), polyamide materials (PA12/Nylon), high-performance polymers PSU, PVDF, PTFE, PEEK, and ceramic materials (zirconia, alumina). These materials have undergone comprehensive biocompatibility testing and received approval for use in medical devices, pharmaceutical processing equipment, or clinical applications involving direct or indirect patient contact.

The successful qualification of these materials for medical use reflects their chemical inertness, absence of leachable toxic compounds, and compatibility with medical sterilization protocols. PVC, PE, and PP are widely deployed in disposable medical devices, while PTFE and PVDF serve in implantable devices and critical pharmaceutical processing applications. Ceramics are established materials for orthopedic and dental implants, demonstrating exceptional biocompatibility and chemical stability. The medical grade designation positions these materials as optimal candidates for membrane distillation applications in pharmaceutical water purification, sterile concentrate production, blood fraction processing, and other clinical or pharmaceutical scenarios requiring the highest material purity and biocompatibility standards.

Materials classified as "False" for medical grade certification include PS, SBS, CN, and CA. While these materials may be safe for general consumer applications and potentially approved for food contact (PS, SBS), they lack the comprehensive biocompatibility testing, extractables/leachables characterization, and regulatory submissions required for medical device designation. The exclusion of these materials from medical grade classification reflects either insufficient regulatory investment by manufacturers to pursue medical device approval, presence of chemical constituents or processing residuals that fail biocompatibility testing, or material property limitations (thermal stability, sterilization compatibility) incompatible with medical device requirements. These materials are precluded from deployment in pharmaceutical water production, parenteral solution preparation, blood processing, or any membrane distillation application where the product stream contacts patients or pharmaceutical formulations intended for injection.

The regulatory compliance profiles reveal critical insights for membrane material selection in specialized high-value applications. The broad food contact spanning commodity polymers,

high-performance polymers, bioplastics, and ceramics provides substantial material selection flexibility for food processing, beverage production, and potable water applications, enabling performance optimization without regulatory constraints. The narrower subset of materials achieving medical grade certification imposes more restrictive selection criteria for pharmaceutical and clinical applications, though the qualification of key high-performance polymers including PVDF, PTFE, PEEK and ceramics ensures availability of materials with exceptional chemical resistance, thermal stability, and vapor transport properties suitable for demanding pharmaceutical processing environments. The overlap between food contact approval and medical grade certification is substantial but not complete. Materials approved for food contact but lacking medical grade certification such as PS, SBS, CN, CA may serve in food processing or potable water applications but cannot be deployed in pharmaceutical or clinical scenarios.

For membrane distillation applications in the circular water economy involving resource recovery from food processing waste streams, the universal food contact approval of ceramics and fluoropolymers: PVDF, PTFE, ECTFE, PEEK provides regulatory certainty alongside their demonstrated chemical resistance, thermal stability, and pH tolerance. These materials enable deployment in complex industrial scenarios where recovered water or concentrated products may re-enter food production streams, pharmaceutical manufacturing, or potable water supplies, ensuring both technical performance and regulatory compliance. The medical grade certification of PVDF, PTFE, PEEK, and ceramics further positions these materials as universal candidates suitable for the most stringent regulatory environments, from medical device manufacturing and pharmaceutical production to potable water supply and food processing, representing optimal choices for applications requiring maximum regulatory assurance alongside superior technical performance.

S3. End-of-life Energy and CO_2 Offset Potential

The membranes end of life potential was also calculated based on their respective recycling efficiency reported in the literature, the energy offset, and CO2 emission offset values calculated using Ecoaudit tool is shown in Table S2. Out of the top four energy intensive materials, PEEK has the highest energy offset followed by PES, PI and PTFE. While CN has significantly high energy offset despite being the lowest energy intensive material compared to all the other reported materials. PEEK also has the highest CO_2 emission offset compared to the top four CO_2 emitters and for the materials with the lowest CO_2 footprint, PS has the highest offset followed by PE, PP, and PLA.

Table S2. End-of-Life (EOL) Energy Recovery and CO_2 Emission Offset Potential for Candidate Membrane Materials with Reported Recycling Efficiencies.

**Material Class**	**Membrane Material**	**Energy offset ($\times 10^4$ MJ, EOL recycling)**	**CO_2 emission offset ($\times 10^2$ kg, EOL recycling)**	**% Recovery (Literature)**
Plastics	PTFE	-1.1	-8.9	88.3 [54]
	PVDF	-5.3	-49.0	89.2 [55]
	PSU	-6.5	-35.0	99 [56]
	PP	-2.6	-6.8	99.9 [57]
	PES	-7.1	-38.0	99 [56]
	ABS	-3.3	-13.0	99 [58]
	SBS	-3.6	-17.0	99 [58]
	SAN	-3.3	-12.0	90 [59]

	PVC	-1.8	-6.4	99 [60]
	PS	-2.9	-9.3	86 [61]
	PLA	-3.2	-1.7	95 [62]
	PI	-1.8	-9.8	85 [63]
	ECTFE	-3.6	-18.0	88.3 [54]
	Nylon	-4.0	-26	93 [64]
	PEEK	-1.0	-57	85 [65]
	PET	-2.8	-12	95 [66]
	PE	-2.8	-7.6	90 [67]
Bioplastics	CN	-0.7	-3.6	90 [68]
	CA	-0.6	-2.1	90 [69]
Ceramics	Alumina	-0.5	-2.6	99 [70]
	Zirconia	-0.6	-3.9	99 [70]
	Titania	-3.1	-19	99 [70]

Negative values indicate energy recovery or emission offsets from recycling processes

S4. Custom Designed Membrane Distillation Setup

The membrane distillation performance of three commercially available membranes, PVDF (ε~0.62), PTFE (ε~0.77), and PP(ε~0.6) with pore size of 0.45 μm and thickness of 200 μm obtained from Tisch Scientific were tested using a direct contact membrane distillation, the system comprises of a custom MD module shown in Figure S2 that hosts the hydrophobic membrane with active membrane area of 4 cm^2. The hydrophobic membrane separates the hot feed and cold permeate maintained at 70 °C ± 1°C and 20 °C ± 1°C respectively using water heater and chiller from Julabo, USA. Two peristaltic pumps from Knf, Germany were used to recirculate both the

hot feed and cold permeate with a flow rate of 100ml/min. 35 g/L NaCl solution and distilled water were used as the feed and permeate, respectively. The weight and electrical conductivity of the permeate collected over time was measured and recorded using a weigh scale (Mettler Toledo, USA) and conductivity meter (Hannah Instruments, USA), respectively every five minutes.

Salt rejection efficiency over 36 h of MD was determined using:

$$R = \left(1 - \frac{C_p}{C_f}\right) \times 100 \qquad (S1)$$

where C_f and C_p represent feed and permeate concentrations, respectively.

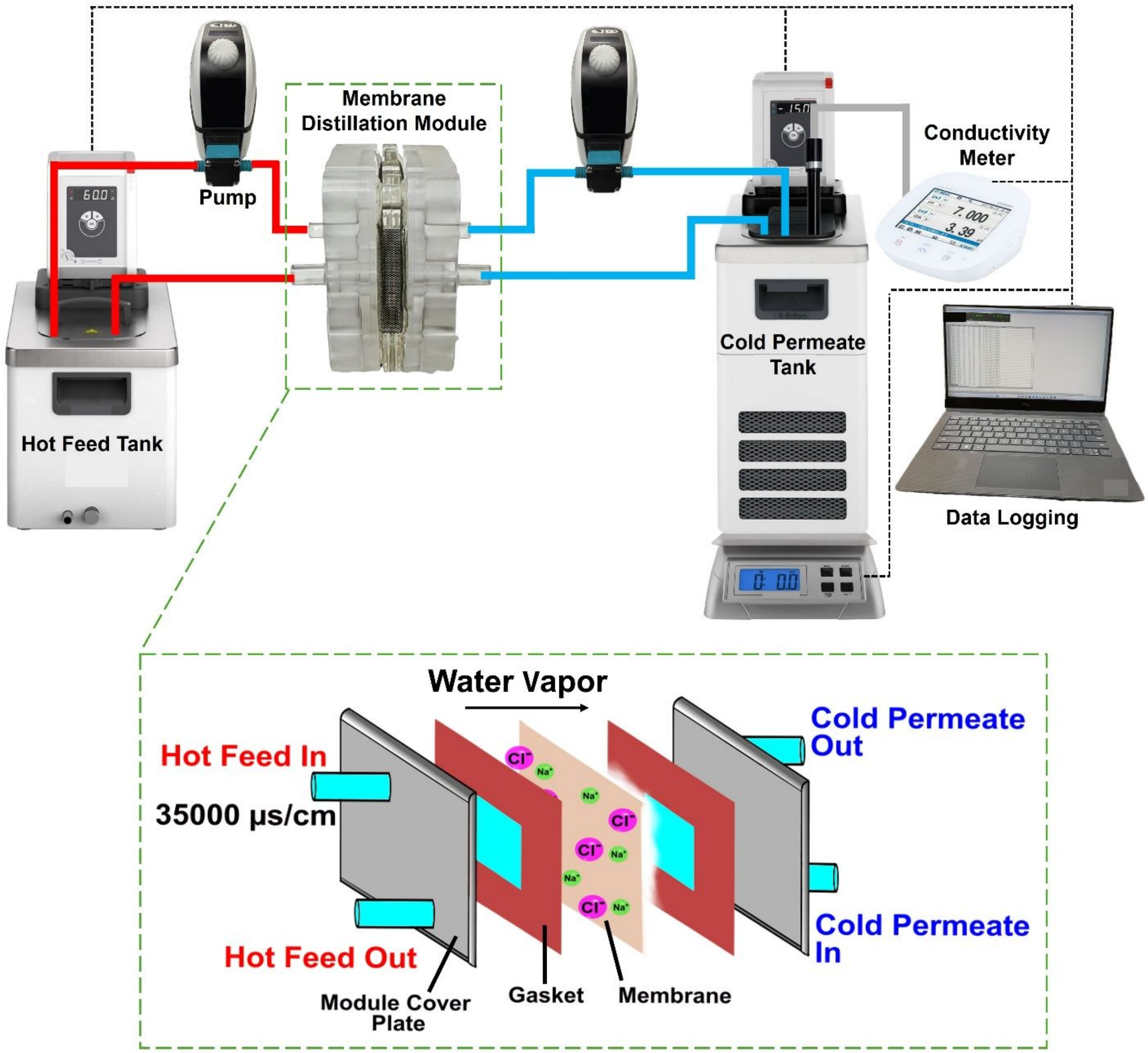

Figure S2. Schematic diagram of a direct contact membrane distillation process (DCMD).

S5. Calculations of Material Performance Analysis

The following assumptions were made in water vapor flux prediction model; each fabricated membranes are assumed to be engineering with porosity of 0.7 and thickness of 200 µm. The pressure differential between the feed at 70 °C and permeate at 20 °C is calculated

based on Antoine's equation[1]. This equation facilitates obtaining vapor pressure based on the absolute temperature of water on each side is given by:

$$p = \exp\left(23.1964 - \frac{3816.44}{T - 46.13}\right) \quad (S2)$$

The normalization of the parameters is done using the following equations. For parameter which positively contributes to the performance index,

$$x_{norm} = \frac{x - x_{min}}{x_{max} - x_{min}} \quad (S3)$$

For parameter which negatively contributes to the performance index,

$$y_{norm} = \frac{y_{max} - y}{y_{max} - y_{min}} \quad (S4)$$

This normalization approach ensures that the performance index remains greater than zero when applied in Equation 3. The value of weights for these different criteria is shown in Table S3.

Table S3. Values of weights chosen as per the criteria

	ω_{Tmax}	ω_{σ}	ω_{Wv}	ω_{k}	ω_{S}
Equal importance	0.2	0.2	0.2	0.2	0.2
High flux priority	0.1	0.1	0.6	0.1	0.1
Sustainability Priority	0.1	0.1	0.1	0.1	0.6

Table S4. Performance Index Parameter Normalization Direction and Rationale

Parameter	Normalization	Rationale
T_max	Positive (Eq. S2)	Higher is better
σ	Positive (Eq. S2)	Higher is better
W_v	Positive (Eq. S2)	Higher is better
k	**Negative (Eq. S3)**	**Lower is better**
E	Negative (Eq. S3)	Lower is better
CO_2	Negative (Eq. S3)	Lower is better

The performance index (Π) framework employed min-max normalization across all 22 candidate materials, whereby each material's property value is scaled relative to the minimum and maximum observed within the full material pool. Because SBS exhibits an anomalously high intrinsic water vapor transmission rate (W_v~ ≈ 310 g·mm·m^{-2}·day^{-1}) approximately 1.5 times that of the next-highest material (PSU) and over an order of magnitude above the median polymer value its inclusion as the normalization maximum compresses the relative W_v scores of all other materials. To assess whether this statistical outlier effect disproportionately distorts Π rankings for commonly deployed MD polymers (PE, PVDF, PP, PTFE), a sensitivity analysis was conducted by recalculating Π under the equal-weighting scenario with SBS excluded from the normalization pool. All other normalization boundaries, weighting factors, and property parameters were held constantly. Table S5 reports Π values for all 22 materials under both the full-pool (original) and SBS-excluded normalization conditions, along with the resulting rank changes. The analysis reveals that ceramic materials (Titania, Zirconia) and most commodity polymers are insensitive to SBS exclusion, with rank changes of 0–2 positions. The most notable redistribution occurs for PSU, which advances 11 rank positions (Π = 0.37 → 0.55) when SBS is excluded, reflecting PSU's

second-highest W_v among the remaining 21 materials. Under flux-priority weighting with SBS excluded, PSU emerges as the top-ranked polymer ($\Pi = 0.775$), confirming it as the most practical high-flux alternative to SBS for MD applications. The robustness of ceramic rankings (Titania $\Pi = 0.67$ unchanged; Zirconia $\Pi = 0.63$ unchanged) and sustainability-priority rankings under both normalization conditions confirm that the framework's principal recommendations are not artifacts of SBS's outlier vapor transmission value.

Table S5. Performance Index (Π) sensitivity analysis under equal weighting: comparison of rankings with SBS included in (full normalization pool, n = 22) versus excluded from (SBS-excluded pool, n = 21) the min-max normalization boundary. Rank change is calculated as rank in full pool- rank in SBS excluded pool; positive values indicate improvement when SBS is removed. N/A entries for SBS reflect its removal from the comparison pool. Under flux-priority weighting with SBS excluded, PSU achieves $\Pi = 0.775$ and becomes the top-ranked polymer candidate

**Material**	**Π (Equal, Full)**	**Π (Equal, excl. SBS)**	**Rank Change**
Titania	0.67	0.67	0
Zirconia	0.63	0.63	0
SBS	0.55	N/A	N/A
Alumina	0.46	0.46	1
PSU	0.37	0.55	11
PVDF	0.29	0.30	2

S6. Economic Feasibility and Cost-Performance Trade-offs

Economic viability represents a critical dimension of material selection decisions, particularly in industrial-scale deployment where material costs accumulate proportionally to production volume and operational timeframe. Material pricing data extracted from Ansys Granta provides a systematic basis for evaluating the cost implications of various material selections and identifying potential economic constraints on technology adoption shown in Figure S3. Analysis of material costs across the evaluated portfolio reveals substantial variation in unit pricing, reflecting fundamental differences in synthesis complexity, production scale, and market maturity. PEEK, (ECTFE), and ceramic materials including zirconia represent the premium cost tier, each commanding prices exceeding 25 USD/kg. These high-performance materials, despite their exceptional thermal stability, mechanical strength, and chemical durability, face significant economic barriers to their adaptation in cost-sensitive applications. The elevated material costs of ceramic alternatives, while justified by their superior performance characteristics in aggressive operational environments, may restrict their utilization to specialized applications where performance requirements justify the economic premium. In marked contrast, conventional thermoplastic polymers demonstrate substantially lower unit costs, positioning them as economically attractive candidates for general-purpose applications. PE, PET, and PVC exemplify this cost-effective category, each priced below 5 USD/kg and offering favorable performance characteristics for standard membrane distillation operation. Bioplastic materials occupy an intermediate cost position between conventional polymers and ceramics, with pricing generally ranging from 4 to 6 USD/kg. This economic stratification establishes a fundamental performance-cost trade-off inherent in material selection. While ceramic and high-performance polymeric membranes deliver superior thermal stability, mechanical robustness, and chemical resistance

critical for demanding industrial environments, their elevated material costs necessitate rigorous cost-benefit analysis to justify deployment. Conversely, economically accessible conventional polymers provide adequate performance for most moderate-severity applications but may exhibit limitations under extreme operational conditions. Optimization of membrane material selection therefore requires balancing technical performance requirements against economic constraints, ensuring that selected materials deliver requisite functionality at acceptable cost levels compatible with project economics and industry competitiveness. For applications involving harsh chemical environments or extended operational lifespans where performance superiority translates to extended membrane durability and reduced replacement frequency, the economic premium associated with high-performance materials may be justified through lower total cost of ownership. For standard desalination and wastewater treatment applications with moderate operational demands, cost-effective polymeric alternatives provide appropriate performance-to-cost optimization.

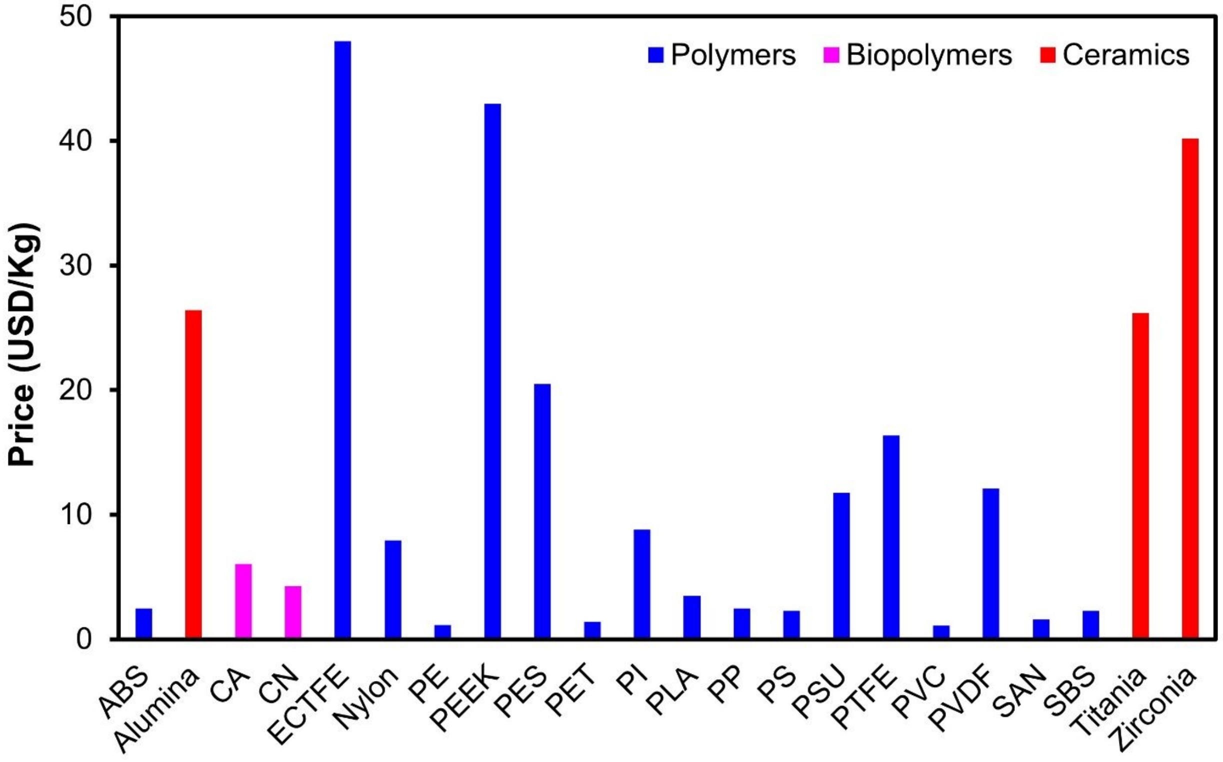

Figure S3. Material cost distribution across candidate membrane materials (USD/kg) from Ansys Granta database, illustrating distinct economic stratification: high-cost premium materials (PEEK, ECTFE, zirconia >25 USD/kg), intermediate-cost ceramics and some polymers (alumina, titania ~5-26 USD/kg), and cost-effective conventional thermoplastics and bioplastics (<5 USD/kg), demonstrating fundamental performance-cost trade-offs in material selection decisions.

References

(1) Thomson, G. W. The Antoine equation for vapor-pressure data. *Chemical reviews* **1946**, *38* (1), 1–39.
